# Nanoconfinement-Driven Solid-State Ratiometric Fluorescent Aptasensor for 17β-Estradiol Detection in Complex Matrices

**DOI:** 10.3390/bios16080419

**Published:** 2026-08-03

**Authors:** Shanshan Zheng, Hui Wang, Zhixue Yu, Ruipeng Chen, Liang Yang, Benhai Xiong, Xiangfang Tang

**Affiliations:** State Key Laboratory of Animal Nutrition and Feeding, Institute of Animal Science, Chinese Academy of Agricultural Sciences, Beijing 100193, China; zhengshanshan@caas.cn (S.Z.);

**Keywords:** 17β-estradiol, ratiometric fluorescent aptasensor, nanoconfinement, anodic aluminum oxide (AAO), complex matrices

## Abstract

Precise quantitative monitoring of 17β-estradiol (E2) is important for reproductive management in precision livestock farming. However, E2 determination in complex biological matrices remains challenging because of matrix-derived background and signal variability. Here, we developed a nanoconfinement-assisted solid-state ratiometric fluorescent aptasensor integrating target-induced strand displacement (TISD), magnetic separation, and anodic aluminum oxide (AAO) nanochannel confinement. The sensing probe consisted of streptavidin-coated magnetic nanoparticles (MNPs) carrying a FAM-labeled cDNA internal reference and a Texas Red-labeled E2 aptamer reporter. E2 binding promoted dissociation of the Texas Red-labeled aptamer from the magnetic probe. Magnetic separation and washing reduced soluble matrix-derived interference, while subsequent deposition of the sensing complexes onto an AAO membrane mitigated coffee-ring-associated nonuniformity and produced a more spatially uniform dual-color fluorescence distribution for ratiometric analysis. Under matrix-matched calibration conditions, linear ranges of 5.0–50.0 pM were obtained in tap water and sow saliva, 5.0–40.0 pM in whole milk, and 5.0–15.0 pM in post-estrus sow urine. The LOD determined in tap water was 3.62 pM. The different calibration slopes obtained among the four matrices indicated that residual matrix-dependent effects remained and that matrix-specific calibration was required for quantitative analysis. Matrix-matched spike recoveries ranged from 86.92% to 119.54% across the investigated matrices. The aptasensor exhibited the strongest response toward 17β-E2 among the tested compounds; however, cross-reactivities of 77.3% for E3 and 47.3% for 17α-E2 indicated preferential rather than exclusive recognition. Molecular docking suggested a putative binding pose but did not experimentally establish the molecular recognition mechanism. Overall, the platform demonstrated laboratory-scale analytical feasibility in pretreated tap water, sow saliva, whole milk, and post-estrus sow urine. Further development of sample preparation, magnetic handling, membrane loading, probe selectivity, and portable fluorescence readout will be required before in situ or on-site application.

## 1. Introduction

Precision livestock farming integrates sensing technologies, connected devices, and data analysis to support the monitoring and management of individual animals [1]. Reproductive monitoring is an important component of this framework because inaccurate identification of estrus can delay insemination, prolong the reproductive cycle, and increase production costs. In dairy production, for example, manual observation is labor intensive and may fail to detect animals with weak or silent behavioral signs [2,3]. Although activity, temperature, and movement sensors have improved estrus detection, their performance may still be affected by individual behavior, housing conditions, and data-integration methods [4,5]. Measurement of reproductive hormones provides complementary physiological information that is less directly dependent on observable behavior. Among these hormones, 17β-estradiol (E2) is an important biomarker associated with follicular development and estrus-related physiological changes in livestock [6,7].

Conventional methods for E2 determination include enzyme-linked immunosorbent assay (ELISA) and liquid chromatography–tandem mass spectrometry (LC–MS/MS). ELISA is relatively accessible but may be affected by antibody cross-reactivity and matrix-dependent interference, whereas LC–MS/MS provides high analytical selectivity but requires specialized instrumentation, trained personnel, and extensive sample preparation [8,9].

These limitations have encouraged the development of biosensing approaches suitable for simplified E2 analysis. Aptamers are synthetic single-stranded nucleic acids selected for target binding and offer several practical advantages, including chemical synthesis, sequence modification, and compatibility with fluorescence labeling [10,11,12,13]. However, conventional single-channel fluorescent aptasensors remain susceptible to nonspecific background signals and variations in excitation intensity, probe concentration, sample volume, and imaging conditions, particularly in complex matrices such as milk, saliva, and urine [14,15,16].

Ratiometric fluorescent biosensors measure the relative responses of two fluorescence channels, typically using one signal as a target-responsive reporter and the other as an internal reference [17,18]. Because the two signals are acquired under comparable experimental conditions, their ratio can partially compensate for variations in illumination, probe loading, sample handling, and detector response. Ratiometric fluorescence strategies have consequently been applied to E2 determination. For example, a ratiometric upconversion-luminescence aptasensing platform integrated with a paper-based device and smartphone imaging was developed for 17β-E2 analysis [19]. Many previously reported ratiometric E2 platforms primarily provide spectral self-calibration through solution- or paper-based fluorescence readout but do not specifically control the spatial redistribution of particle-associated probes during solid-state fluorescence collection. The AAO-assisted step introduced in the present study was intended to localize the magnetically isolated sensing complexes within a defined detection area and reduce evaporation-driven edge accumulation and spatial fluorescence heterogeneity.

Magnetic nanomaterials can serve as probe carriers and separation materials, enabling target-associated sensing complexes to be isolated from soluble matrix components before fluorescence measurement [20]. However, when particle-containing droplets are deposited on solid substrates, contact-line pinning and evaporation-driven outward capillary flow can transport suspended materials toward the droplet edge, producing a coffee-ring deposit and a nonuniform signal distribution [21,22].

Nanoporous anodic alumina, also referred to as anodic aluminum oxide (AAO), possesses an ordered array of vertically oriented nanopores and has been widely investigated as a substrate for optical biosensing [23]. The pore geometry of AAO can promote downward capillary flow during droplet drying and, under appropriate conditions, reduce the outward transport and edge accumulation of deposited materials [24]. Recent reviews and experimental studies have further demonstrated the applicability of NAA/AAO substrates in optical and fluorescence-based biosensing [25,26]. On this basis, the present study investigated whether a commercial AAO membrane could improve the spatial uniformity of particle-associated fluorescence and mitigate coffee-ring formation in the proposed solid-state assay.

In this study, a solid-state ratiometric fluorescent aptasensor was developed by integrating target-induced strand displacement, magnetic separation, and AAO nanochannel confinement. A FAM-labeled complementary DNA strand immobilized on streptavidin-coated magnetic nanoparticles served as the internal reference, while a Texas Red-labeled E2 aptamer functioned as the target-responsive reporter. E2 binding promoted dissociation of the reporter aptamer from the magnetic probe, after which magnetic separation reduced soluble matrix-derived interference. The remaining sensing complexes were deposited onto an AAO membrane to improve the uniformity of solid-state fluorescence imaging. The analytical response, matrix dependence, selectivity, preliminary reliability, and applicability of the platform were evaluated using tap water, sow saliva, whole milk, and post-estrus sow urine. The study was intended to assess the laboratory-scale feasibility and limitations of combining magnetic handling, ratiometric fluorescence, and AAO-assisted solid-state readout for E2 determination in pretreated complex matrices.

## 2. Materials and Methods

### 2.1. Reagents and Materials

All chemicals and reagents were of analytical grade and used as received without further purification. 17β-Estradiol (E2, 99.5%), estriol (E3, 98%), 17α-estradiol (98%), bisphenol A (BPA, >99.0%), poly-L-lysine hydrobromide (MW: 150,000–300,000), and magnesium chloride hexahydrate (MgCl_2_· 6H_2_O, ≥99.0%) were obtained from Macklin Biochemical Co., Ltd. (Shanghai, China). Anodic aluminum oxide (AAO) membranes (pore diameter: 0.2 µm; diameter: 13 mm) were sourced from Whatman (Maidstone, UK), with quartz slides (25 mm × 75 mm × 1 mm) used as planar control substrates. Streptavidin-coated magnetic beads (1 µm diameter; binding capacity ≥ 450 pmol/mg) were employed for probe immobilization (BEAVER, Suzhou, China).

The buffer solution (Mg-PBST) consisted of 1× phosphate-buffered saline (PBS) supplemented with 0.05% Tween-20 and 5 mM MgCl_2_ (pH 7.4). This buffer was prepared weekly and stored at 4 °C. Phosphate-buffered saline with Tween-20 (PBS-T) was prepared using PBS-T fast tablets (Macklin). Ultrapure water (18.2 MΩ· cm) was provided by a Milli-Q system (Merck Millipore, Darmstadt, Germany).

The Texas Red-labeled aptamer and the dual-labeled (FAM and biotin) complementary DNA (cDNA) were synthesized and HPLC-purified by Sangon Biotech Co., Ltd. (Shanghai, China). The aptamer sequence corresponds to the Alsager 35-mer aptamer as reported by Svobodová et al. [12]. The detailed sequences and modifications are summarized in Table 1.

### 2.2. Instrumentation

The surface morphology of the functionalized magnetic nanoparticles and the AAO membranes was characterized using a field-emission scanning electron microscope (FE-SEM; SU8020, Hitachi, Tokyo, Japan). To further investigate the internal structure and nanoscale dimension of the magnetic nanoparticles, transmission electron microscopy (TEM) was performed using a FEI Tecnai G2 F30 microscope (HRTEM, Waltham, MA, USA). Additionally, the surface chemical modifications and structural fingerprints of the functionalized probes were verified by Raman spectroscopy using a LabRAM HR Evolution Raman spectrometer (Horiba, Kyoto, Japan) equipped with a 532 nm laser excitation source. Fluorescence imaging was conducted on a Leica DM6000B upright fluorescence microscope (Leica Microsystems, Wetzlar, Germany) equipped with a CCD camera and various plan semi-apochromatic objectives. To ensure precise ratiometric detection, a GFP filter set (Ex: 450–490 nm, Em: 500–550 nm) and an N2.2.1 filter set (Ex: 515–560 nm, Em: ≥590 nm) were employed for the FAM and Texas Red channels, respectively. Image acquisition was managed via LAS X (version 3.7.4; Leica Microsystems, Wetzlar, Germany), and subsequent quantitative analysis was performed using ImageJ software (version 1.54P, NIH, Bethesda, MD, USA).

### 2.3. Preparation of Dual-Fluorescent Probes

The dual-fluorescent ratiometric probe, comprising magnetic nanoparticles (MNPs) functionalized with a FAM-labeled complementary DNA (cDNA-FAM) and a Texas Red-labeled aptamer, was synthesized through a two-step solid-phase assembly process. The immobilization of the internal reference strand was performed as follows: An aliquot of 100 µL of MNPs was magnetically separated, and the supernatant was discarded. The beads were washed three times with the working buffer (Mg-PBST, pH 7.4) to remove storage salts and preservatives. Subsequently, 12 µL of cDNA-FAM stock solution (100 µM) was added to the MNP suspension, and the total volume was adjusted to 150 µL with the buffer. The mixture was incubated on a thermomixer (e.g., 1000 rpm, 20 °C) for 1 h at room temperature in the dark to ensure efficient anchoring of the cDNA-FAM onto the MNP surface. Following incubation, the functionalized MNPs (MNP-cDNA-FAM) were isolated via magnetic decantation and rinsed three times with the buffer to ensure the complete removal of any unbound oligonucleotides. The pooled supernatants and washing fractions collected during this immobilization step were retained for fluorescence-based quantification of unbound cDNA-FAM. The fluorescence intensity was measured under identical conditions, and a calibration curve established using free cDNA-FAM was used to convert fluorescence signals into cDNA-FAM amounts. The immobilization efficiency was calculated by fluorescence mass balance as the ratio of immobilized cDNA-FAM amount to the total amount initially added.

Next, a low-temperature, stoichiometry-controlled hybridization strategy was employed to assemble the reporter probe. To minimize the non-specific background and maximize the target-induced signal quenching efficiency (the “starvation” strategy), a strictly optimized volume (3.5 μL) of the Texas Red-labeled aptamer (100 μM) was introduced into the MNP-cDNA-FAM suspension (total volume 150 μL). To promote thermodynamically favorable and highly specific double-strand formation while preserving the photostability of the fluorophores, the mixture was securely sealed to prevent condensation and incubated in a thermomixer at 4 °C overnight (12–16 h) under gentle agitation (1000 rpm) in complete darkness. Following hybridization, the resultant dual-color ratiometric probes (MNP-cDNA/Apt) were collected via magnetic separation, washed three times with the working buffer to remove any unhybridized aptamers, and finally resuspended in a final volume of 100 μL of the working buffer, matching the initial MNP sampling volume. The post-hybridization supernatant and the three initial washing fractions were collected and pooled for fluorescence-based quantification of unbound Texas Red-labeled aptamer. The fluorescence signal was converted into aptamer amount using a calibration curve prepared with free Texas Red-labeled aptamer under identical measurement conditions. The amount retained on the MNP–cDNA complexes after these initial washing steps was calculated by subtracting the measured unbound amount from the total amount initially added. Because the subsequent thermal-equilibration step and five stringent washing steps were not included in this fluorescence mass-balance measurement, the resulting value is referred to as the apparent post-hybridization retention efficiency rather than the final aptamer-loading efficiency of the ready-to-use probe.

Finally, a thermal equilibration and stringent washing protocol was applied to eliminate weakly bound or mismatched sequences. Prior to use, the overnight-incubated mixture was transferred to a 20 °C environment and subjected to continuous shaking for 30–45 min. This equilibration step was strategically designed to induce the dissociation of non-specifically adsorbed or thermodynamically unstable aptamers. Following equilibration, the complexes were immediately collected via magnetic separation and rigorously washed five times with the room-temperature working buffer to ensure maximum probe purity. The resulting dual-fluorescent probes were resuspended in 15 μL of the working buffer and immediately employed for the subsequent target recognition assays to ensure optimal sensing performance. The aptamer released during these five stringent washing steps was not separately quantified. Therefore, the exact Texas Red-aptamer loading remaining on the final ready-to-use probe was not directly determined in the present study.

### 2.4. Sensing Procedure for 17β-E2

The quantitative detection of 17β-E2 was executed through a competitive target-induced strand displacement (TISD) assay, followed by solid-state dual-fluorescence imaging on AAO nanochannel arrays. First, the target recognition and competitive displacement reaction were initiated. In a typical assay, the reaction was performed in a low-binding microcentrifuge tube to minimize non-specific adsorption of the target and probes. A 100 µL reaction system was prepared, comprising 10 µL of the freshly prepared dual-fluorescent MNP probe, 10 µL of 17β-E2 standard solutions (or actual water samples) at varying concentrations, and 80 µL of the binding buffer (Mg-PBST, pH 7.4). The mixture was incubated in a thermomixer at 20 °C with continuous agitation at 1000 rpm for 45 min in the dark. This incubation period was selected on the basis of the time-optimization experiment and allowed reproducible development of the target-responsive signal.

Second, rapid magnetic separation and sensing-complex purification were performed. Following the 45-min incubation, the tubes were briefly centrifuged for 3 s to collect any condensation from the tube caps. The intact MNP–cDNA–FAM complexes (retaining the internal reference signal and residual reporter probes) were rapidly isolated using a magnetic separation rack, while the target-displaced Texas Red-labeled aptamer fragments were removed with the supernatant. To reduce residual matrix interference, the complexes were gently washed twice with 100 μL of binding buffer. After washing, the purified sensing complexes were resuspended in 15 μL of binding buffer for subsequent solid-state fluorescence analysis.

Finally, solid-state fluorescence imaging and ratiometric analysis were conducted. A 10 μL aliquot of the purified sensing suspension was carefully deposited onto a highly ordered AAO membrane (13 mm diameter) positioned on a standard glass slide. To minimize the coffee-ring effect during evaporation and improve fluorescence distribution uniformity, the deposited membrane was immediately covered with an 18 × 18 mm square microscope coverslip using a bubble-free oblique dropping technique. This confined fluidic layer reduced variations in sample geometry during drying and imaging, thereby improving the reproducibility of fluorescence acquisition. The dual-color fluorescence images were acquired using a fluorescence microscope equipped with standardized green (FAM) and red (Texas Red) filter sets. To ensure consistent quantitative imaging, the excitation intensity, exposure time, and detector gain parameters were strictly maintained across all samples. The digitized grayscale intensities of the target regions were extracted using ImageJ software (version 1.54P). To minimize background fluctuations and enhance analytical reliability, the baseline-subtracted relative signal variation, defined as Δ*R* (Δ*R* = R_0_—R, where R = I_Red_/I_Green_), was calculated. Here, R_0_ and Ratio represent the fluorescence intensity ratios in the absence and presence of E2, respectively. Because E2 binding caused detachment of the red fluorophore-labeled aptamer from the magnetic complexes, the I_Red_/I_Green_ ratio decreased upon target recognition, resulting in a positive Δ*R* value that increased with increasing E2 concentration. Finally, Δ*R* was plotted against the logarithmic E2 concentration to construct the calibration curve.

The present analytical workflow was performed manually under laboratory conditions. Although the AAO membranes were commercially obtained rather than fabricated in-house, sample deposition onto the membrane, coverslip placement, magnetic separation, washing, fluorescence imaging, and image analysis were manually conducted. The sensing workflow required a 45-min target-recognition step followed by magnetic separation, two washing steps, AAO loading, and dual-channel fluorescence acquisition. Matrix-specific sample pretreatment was performed separately and was not included in the sensing time.

### 2.5. Optimization of Experimental Conditions

The experimental conditions of the ratiometric fluorescence sensor were optimized using a one-factor-at-a-time approach. The molar ratio of the FAM-labeled probe to the Texas Red-labeled aptamer probe was evaluated at 0.8:1, 1.7:1, 3.4:1, 4.2:1, and 5.8:1. Incubation times of 15, 30, 45, 60, 90, and 120 min and Mg^2+^ concentrations of 2, 5, 10, 15, and 20 mM were evaluated. The E2 concentration was maintained at 50 pM during these optimization experiments.

The effect of pH was evaluated at pH 6.5, 7.0, 7.4, 7.8, and 8.2 using complete Mg-PBST containing 1× PBS, 0.05% Tween-20, and 5 mM MgCl_2_. The pH of the complete buffer was adjusted using dilute HCl or NaOH and verified using a calibrated pH meter. An E2 concentration of 25 pM was used for the pH optimization experiment. During the optimization of each parameter, all other experimental conditions were maintained unchanged.

The net ratiometric response was calculated as Δ*R* = *R*_0_ − *R*_E2_, where *R*_0_ and *R*_E2_ represent the fluorescence ratios measured in the absence and presence of E2, respectively. The optimization experiments were performed as exploratory screening assays using three independently prepared reactions per condition (*n* = 3). Data are presented as mean ± SD. The working condition was selected according to the overall response trend, signal magnitude, and repeatability rather than formal inferential comparison among all tested conditions.

### 2.6. Selectivity and Cross-Reactivity Evaluation

The selectivity and cross-reactivity of the proposed aptasensor were reassessed under analytically relevant concentration conditions. The available potential interferents, including 17α-estradiol (17α-E2), estriol (E3), and bisphenol A (BPA), were individually tested at 50 pM under the optimized sensing conditions. A blank sample without analyte and 50 pM 17β-estradiol were used as the negative and target controls, respectively. All measurements were independently performed using five separately prepared assays (*n* = 5).

To further evaluate selectivity under excess-competitor conditions, 10 pM 17β-E2 was analyzed alone and in the presence of a fivefold molar excess of each interferent. The mixed groups contained 10 pM 17β-E2 together with 50 pM 17α-E2, E3, or BPA. An additional combined-interferent group contained 10 pM 17β-E2 and 50 pM of each of the three interferents. Five independently prepared assays were evaluated for each condition (*n* = 5).

The fluorescence ratio was calculated as $R=I_{Red}/I_{Green}$. For each measurement, the response was expressed as $\Delta R={\bar{R}}_{blank}-R_{sample}$, where ${\bar{R}}_{blank}$ represents the mean fluorescence ratio of the blank controls. Relative cross-reactivity was calculated as: *CR*(%) = Δ*R*_interferent_/Δ*R*_17β−E2_ × 100%.

Statistical differences among the individual analyte groups were evaluated using one-way analysis of variance followed by Tukey’s multiple-comparison test, with *p* < 0.05 considered statistically significant.

### 2.7. Preparation and Spiking of Real Samples

To evaluate the practical applicability and matrix tolerance of the developed ratiometric aptasensor, four real samples were investigated: tap water, sow saliva, whole milk, and post-estrus sow urine. All biological samples were collected in accordance with relevant ethical guidelines and processed immediately or stored at −20 °C until analysis.

Tap Water: Tap water samples were collected from the local municipal water supply. To eliminate solid suspended particulates, the samples were filtered through a 0.22 μm polyethersulfone (PES) microporous membrane. The filtrate was then boiled for 10 min to remove residual chlorine and cooled to room temperature prior to the assay.

Sow saliva: Sow saliva was collected from healthy sows in a fasting state and stored at −20 °C. Prior to analysis, the frozen saliva underwent a single freeze–thaw cycle. Due to the negligible concentration of endogenous 17β-E2 in sow saliva, the sample was diluted 2-fold by mixing with an equal volume of the working buffer. To disrupt the mucin network and induce denaturation, the mixture was heated in a water bath at 95 °C for 10 min. After cooling to room temperature, the sample was centrifuged in a high-speed refrigerated centrifuge at 10,000 rpm for 15 min at 4 °C to precipitate the coagulated mucins and cellular debris, yielding the clear supernatant as the blank matrix.

Whole Milk: To eliminate optical scattering from lipids and steric interference from casein micelles, 5 mL of raw milk was centrifuged at 10,000 rpm for 15 min at 4 °C. The intermediate layer (defatted milk) layer was collected, excluding the upper lipid layer. To precipitate casein, 10% (*v*/*v*) acetic acid was added dropwise to the defatted milk under vortexing until the pH reached approximately 4.6. After a second centrifugation (10,000 rpm for 10 min), the whey supernatant was collected. Finally, the whey was mixed with an equal volume of 2× working buffer (Mg-PBST) to restore a neutral pH, yielding the blank matrix for subsequent assays.

Post-Estrus Sow Urine: Post-estrus sow urine was collected from a local swine farm. The raw urine was centrifuged at 8000 rpm for 10 min to remove insoluble sediments. To minimize matrix interference from urea and high ionic strength salts, and to adjust the high concentration of endogenous 17β-E2 into the linear dynamic range of the sensor, the urine supernatant was diluted 100-fold (1% *v*/*v*) with the working buffer.

Spiking Procedure: For spike-recovery evaluation, aliquots of 17β-E2 standard solutions were added to the corresponding pretreated matrices. Tap water, sow saliva, and whole milk were fortified at final added concentrations of 5, 20, and 40 pM. The 100-fold diluted post-estrus sow urine was fortified at final added concentrations of 5 and 10 pM. Higher urine spiking levels were not evaluated because the final urine calibration model was established over an added-concentration range of 5–15 pM. Three independently prepared spiked samples were analyzed at each concentration level (*n* = 3). The samples were vortex-mixed and analyzed immediately using the standard aptasensor procedure.

Matrix-matched calibration curves were independently established using the corresponding pretreated tap-water, saliva, milk, and urine matrices. Quantitative results for each sample type were calculated using the calibration equation established in the same matrix rather than the calibration equation obtained in buffer or another matrix.

The urine used for the aptasensor–ELISA comparison was collected from non-estrous sows and was obtained from a separate sample batch from the pooled post-estrus sow urine used for the matrix-specific calibration and spike-recovery experiments. A separate saliva sample batch was also used for the paired method comparison.

### 2.8. Comparison with an ELISA Reference Method

To provide a paired orthogonal comparison in biological matrices, a separate batch of urine collected from non-estrous sows and a separate saliva sample batch were analyzed in parallel using the proposed aptasensor and a commercial Human Estradiol Rapid ELISA Kit (EELR003, Thermo Fisher Scientific, Waltham, MA, USA). The non-estrus urine used in this comparison was distinct from the pooled post-estrus sow urine used for the matrix-specific calibration and spike-recovery evaluation.

For each matrix, E2 was added at final concentrations of 150, 250, and 400 pM. Three independently prepared samples were analyzed at each concentration level (*n* = 3). After E2 addition and thorough mixing, each sample was divided into two aliquots. One aliquot was analyzed using the aptasensor after dilution to a concentration within the corresponding established calibration range, whereas the second aliquot was analyzed using ELISA according to the manufacturer’s instructions.

Before aptasensor analysis, the saliva samples were diluted tenfold, resulting in measurement concentrations of 15, 25, and 40 pM. The urine samples containing 150, 250, and 400 pM E2 were diluted 10-, 20-, and 40-fold, respectively, resulting in measurement concentrations of 15, 12.5, and 10 pM. These concentrations were within the established saliva and urine calibration ranges. The concentrations in the original samples were subsequently obtained by multiplying the measured concentrations by the corresponding dilution factors.

Each independently prepared ELISA sample was measured in duplicate wells. The duplicate wells were treated as technical replicates and were averaged to obtain one ELISA result for each independently prepared sample.

Unspiked non-estrus urine and saliva samples were also analyzed using both methods to assess the corresponding endogenous E2 backgrounds. Results below the detection limit of the aptasensor or below the lower quantifiable range of ELISA were reported as <LOD and <LLOQ, respectively, without extrapolation beyond the corresponding calibration ranges. For samples in which the unspiked result was below the relevant quantifiable limit, recovery was calculated from the measured concentration in the fortified sample relative to the nominal added concentration, without assigning an extrapolated numerical value to the unspiked sample. Detailed paired results are provided in Table S7.

### 2.9. Analytical Validation and Statistical Analysis

An independent aptasensor assay was defined as a separately prepared reaction that underwent the complete target-incubation, magnetic-separation, washing, AAO-loading, fluorescence-imaging, and data-processing workflow. Multiple fields of view or regions of interest obtained from the same AAO membrane were averaged to generate one analytical result and were not treated as independent replicates.

Calibration curves were established using five independently prepared assays at each concentration level (*n* = 5). The LOD was determined using ten independently prepared blank tap-water assays (*n* = 10). The detection threshold was calculated as *y*_LOD_ = ${\bar{y}}_{blank}$ + 3*s*_blank_, where ${\bar{y}}_{blank}$ and *s*_blank_ represent the mean and standard deviation of the blank responses, respectively. Because the analytical response was linear with log_10_(*C*_E2_/pM), the LOD concentration was obtained by back-calculation using *C*_LOD_ = 10^(*y*LOD−*b*)/*a*^, where a and b are the slope and intercept of the tap-water calibration equation. Full-precision regression coefficients were used for the calculation.

For each matrix-specific calibration model, the regression equation, coefficient of determination (R^2^), standard error of the slope, and 95% confidence interval of the slope were calculated. Formal residual-versus-fitted analysis and formal homoscedasticity testing were not performed in the present study. Therefore, the randomness of the residuals and the assumption of constant residual variance were not independently verified. The reported regression statistics were consequently interpreted as measures of calibration fit and slope uncertainty rather than as a complete regression-diagnostic validation.

For matrix-matched calibration, an unspiked aliquot of each pretreated matrix was analyzed to establish the corresponding matrix-specific baseline response (*R*_0_). Known concentrations of E2 were then added to separate aliquots of the same pretreated matrix, and the analytical response was calculated as Δ*R* = *R*_0_ − *R*_spiked_. The concentration used for regression represented the nominal added E2 concentration rather than the absolute total E2 concentration in the original sample.

For the post-estrus sow-urine matrix, ELISA analysis indicated an endogenous E2 concentration of approximately 360–380 pM in the raw pooled urine. After 100-fold dilution, approximately 3.6–3.8 pM endogenous E2 remained in all urine aliquots. The unspiked diluted urine was therefore used as the matrix baseline, and the urine calibration described the incremental response to added E2 above this endogenous background. This approach was used for spike-recovery evaluation and was not intended to independently quantify the absolute endogenous E2 concentration in the original urine sample.

The analytical reliability of the proposed aptasensor was evaluated in terms of intra-day precision, inter-day reproducibility, robustness, operational signal stability, component-batch reproducibility, and short-term storage stability. Intra-day precision was assessed using milk samples spiked with 5, 20, and 40 pM E2, with five independent measurements performed at each concentration on the same day. Inter-day reproducibility was evaluated using tap water samples containing 5, 20, and 40 pM E2 on two independent experimental days.

Batch reproducibility of the AAO component was evaluated using two independently prepared AAO membrane batches at 10 pM E2, with three independent membrane measurements for each batch. Variability between magnetic probes was assessed using two independently functionalized MNP probe batches at 20 pM E2, with three independent measurements per batch.

Method robustness was preliminarily assessed at 20 pM E2 in buffer by introducing small variations around the optimized pH, Mg^2+^ concentration, and incubation time while maintaining all other experimental parameters at their optimized values. Operational signal stability was evaluated by repeatedly recording the fluorescence-ratio response of three independently prepared sensing samples at 0, 10, 20, 30, 45, and 60 min after completion of the sensing reaction. The samples were protected from light between measurements.

The short-term storage stability of the assembled magnetic aptamer probe (MNP–aptamer–cDNA) was evaluated after storage at 4 °C in the dark for 0, 5, and 10 days. At each time point, three independently stored probe aliquots were used to detect 20 pM 17β-E2 under the optimized assay conditions (*n* = 3). The response obtained on Day 0 was defined as 100%. All data are presented as mean ± SD, and RSD (%) was calculated as SD/mean × 100%. Detailed validation designs and corresponding statistical results are provided in Tables S1–S6 in the Supporting Information. This experiment assessed the functional stability of the complete sensing probe. The physical stability of the magnetic nanoparticles (e.g., changes in aggregation, hydrodynamic size, ζ potential, and magnetic-separation efficiency) and the molecular integrity or surface retention of the immobilized aptamer were not separately characterized.

### 2.10. Molecular Docking Simulation

The initial DNA model was built using the AlphaFold Server [27], followed by the introduction of a 5′-Texas Red modification. The three-dimensional structure of the small molecule (Compound CID: 5757) was obtained from PubChem in SDF format. Molecular docking between the 5′-Texas Red-modified single-stranded DNA and 17β-estradiol (E2) was performed using AutoDock Vina (v1.1.2, Scripps Research Institute, La Jolla, CA, USA) [28]. The 5′-Texas Red-modified E2 aptamer DNA structure was used as the receptor, converted, hydrogenated, and assigned Gasteiger charges using OpenBabel to generate a PDBQT file for AutoDock Vina. The small molecule was similarly hydrogenated, assigned Gasteiger charges, and converted to PDBQT format. Docking was conducted globally with the modified DNA as the receptor and E2 as the ligand. Binding energies and relative RMSD values were extracted from the Vina output to generate a ranked list of conformations. The top-ranked docking pose with the most favorable AutoDock Vina score was selected for visualization and putative interaction analysis. The docking results were used only to generate a structural hypothesis regarding possible E2–aptamer interactions and were not interpreted as experimental confirmation of the binding mode, residue-level contacts, or binding affinity.

### 2.11. Declaration of Generative AI Use

During the preparation of this work, the authors used DeepSeek for literature searching and preliminary language refinement to improve readability. After using this tool, the authors reviewed and edited the content as needed and take full responsibility for the final content of the publication.

## 3. Results and Discussion

### 3.1. Principle of the Ratiometric Aptasensor

**Figure 1:**
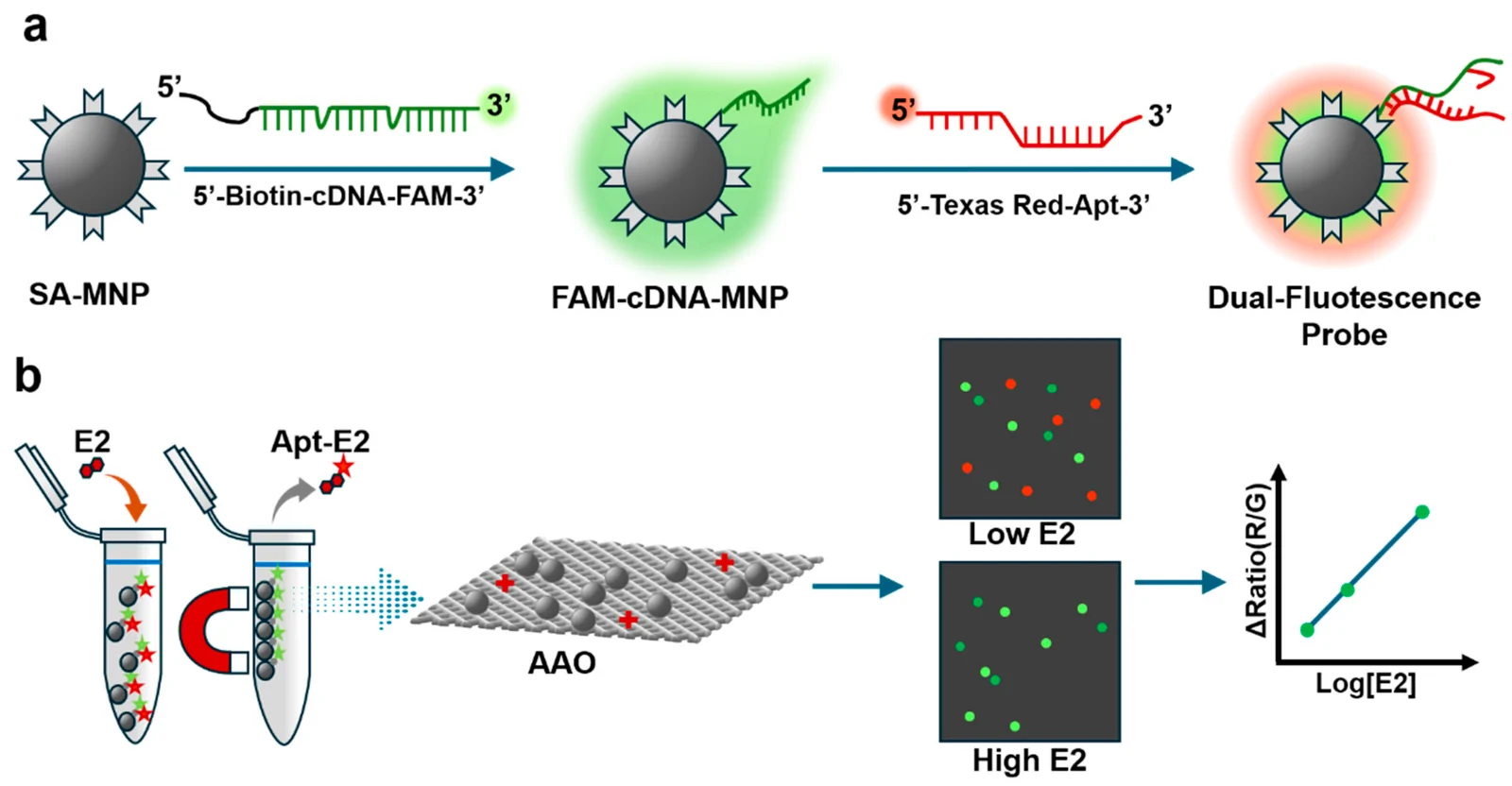
Schematic representation of the sensing strategy: (**a**) Fabrication of the dual-color magnetic probe on SA-MNPs via cDNA-aptamer hybridization, and its specific dissociation upon E2 recognition; (**b**) Nanoconfinement-enhanced fluorescence collection on the AAO membrane for ratiometric detection.

A solid-state dual-color ratiometric sensing strategy was developed based on target-induced strand displacement (TISD), magnetic separation, and anodic aluminum oxide (AAO) nanoconfinement. As illustrated in Figure 1, the dual-fluorescence probe is initially assembled on streptavidin-coated magnetic nanoparticles (SA-MNPs) via the hybridization of a FAM-labeled cDNA (serving as a persistent internal reference with green emission) and a Texas Red-labeled aptamer (acting as a target-responsive reporter with red emission). In the absence of the target, the aptamer completely hybridizes with the cDNA, and the magnetic beads exhibit a strong dual-color fluorescence signal.

Upon the introduction of 17β-estradiol (E2), the aptamer exhibits preferential interaction with E2, triggering a competitive strand displacement. Consequently, the Texas Red-labeled aptamer dissociates from the MNP-cDNA-FAM complex into the supernatant. Following rapid magnetic separation, the displaced Texas Red-labeled aptamer fragments are removed with the supernatant, while subsequent washing reduces residual matrix-derived interferents associated with the sensing complexes. The remaining sensing complexes are then deposited onto an AAO membrane. The spatial confinement provided by the highly ordered nanochannels substantially suppresses the coffee-ring effect and promotes a more uniform dual-color fluorescence distribution. Because the FAM signal remains constant while the Texas Red signal decreases proportionally with the increasing E2 concentration, a reliable ‘turn-off’ ratiometric readout is achieved. This ratiometric design provides a built-in self-calibration mechanism that can partially compensate for variations in experimental conditions.

### 3.2. Characterization of Materials

**Figure 2:**
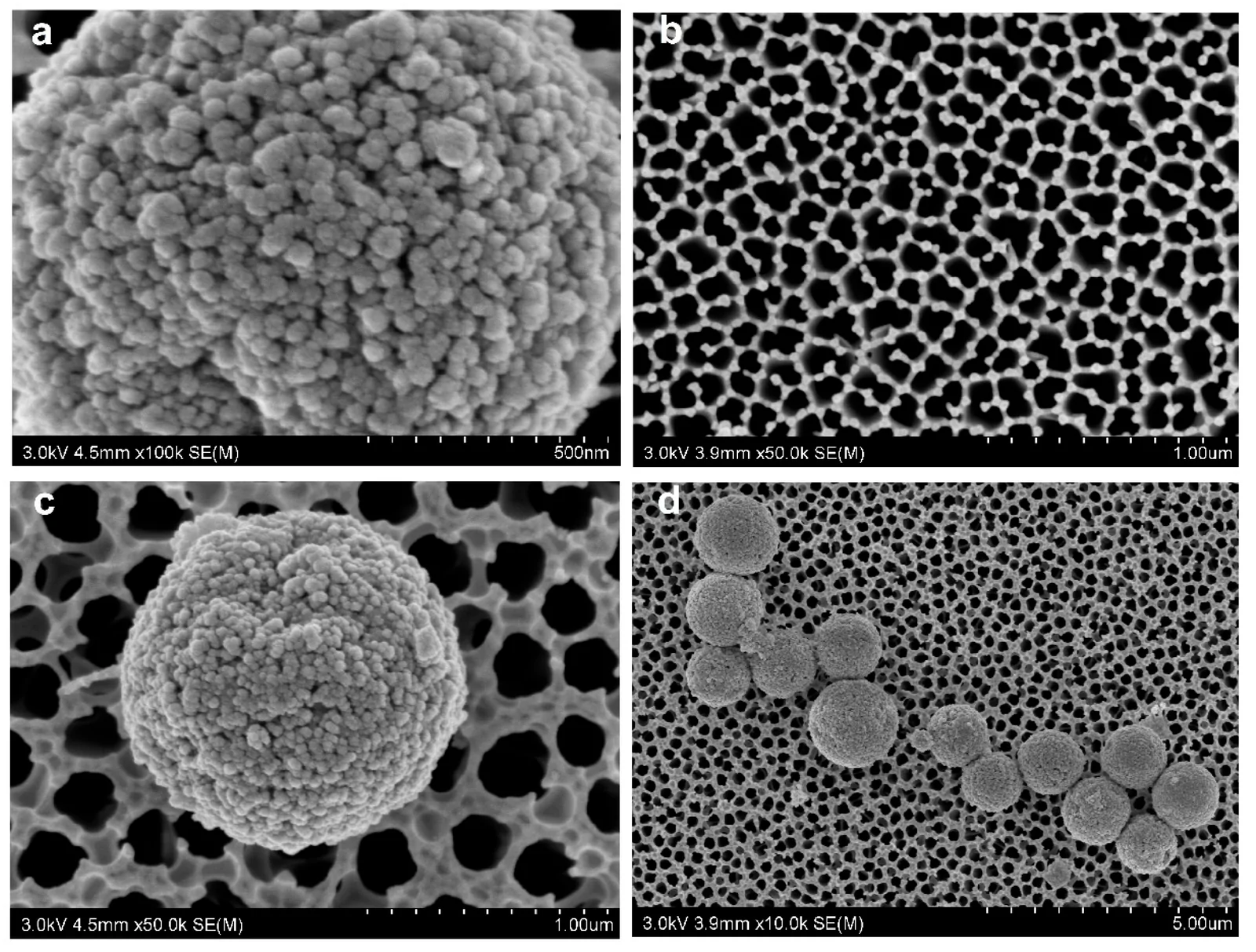
FE-SEM characterization of the solid-state sensing interface. (**a**) Surface morphology of the functionalized MNPs. (**b**) Top-view SEM image of the bare AAO membrane before deposition of the sensing complexes, showing the ordered nanochannel array. (**c**,**d**) MNP-loaded AAO membrane after deposition.

The surface morphologies of the functionalized magnetic nanoparticles (MNPs) and the anodic aluminum oxide (AAO) substrate were characterized to elucidate the spatial confinement mechanism. As shown in Figure 2a, the functionalized MNPs exhibited a uniform spherical morphology with an average diameter of approximately 1 µm. Figure 2b presents a top-view image of the bare AAO membrane (serving as the blank substrate control without any nanoparticle loading), clearly revealing a highly ordered honeycomb-like nanopore array with well-aligned and uniformly distributed channels, and an average pore diameter of approximately 0.2 µm, indicating the good consistency and reproducibility of the AAO substrate. The size difference between the MNPs and the AAO nanopores contributes to a geometric exclusion effect. A high-magnification image (Figure 2c) shows a single MNP located on the membrane surface, indicating that the magnetic probes did not enter the nanochannels. During the droplet evaporation process, the AAO nanochannels serve as vertical fluid drainage paths, and the resulting capillary force drives the buffer solution through the pores. Under these conditions, the lateral particle transport is reduced; this lateral flow typically transports particles to the droplet edge and leads to the coffee-ring effect. Consequently, the sensing complexes are deposited on the AAO surface, forming a relatively uniform and dispersed particle distribution, as illustrated by the multiparticle distribution in Figure 2d. This uniform solid-state distribution contributes to the favorable reproducibility of the dual-color ratiometric fluorescence readout [24].

The spatial confinement effect observed on the AAO membrane can be further understood at the nanoscale structural level. Using depth-profiling transmission electron microscopy, Zhang et al. revealed that the nanochannels in anodic aluminum oxide membranes exhibit a truncated conical geometry along their long axis (approximately 70 µm in length). This shape nonuniformity leads to different reflectance properties along the nanochannels and influences the optical characteristics of the membrane, which provides a physical basis for the capillary-driven vertical drainage and the suppression of lateral particle transport observed in our system [29].

Beyond spatial confinement, AAO substrates have been reported to influence fluorescence behavior in nanoporous systems. Makela, M. et al. demonstrated that nanoporous alumina-based sensors achieve a 15-fold enhancement in fluorescence signal compared to non-porous glass substrates, attributed to the significantly increased surface-to-volume ratio of AAO that provides more immobilization sites for biorecognition elements [30]. Furthermore, Nikitin et al. revealed that Förster resonant energy transfer from anodic alumina luminescence centers to adsorbed dye molecules can provide up to a 5-fold enhancement in dye luminescence, suggesting an additional signal amplification mechanism inherent to AAO substrates that may contribute to the sensitivity of our ratiometric platform [31].

**Figure 3:**
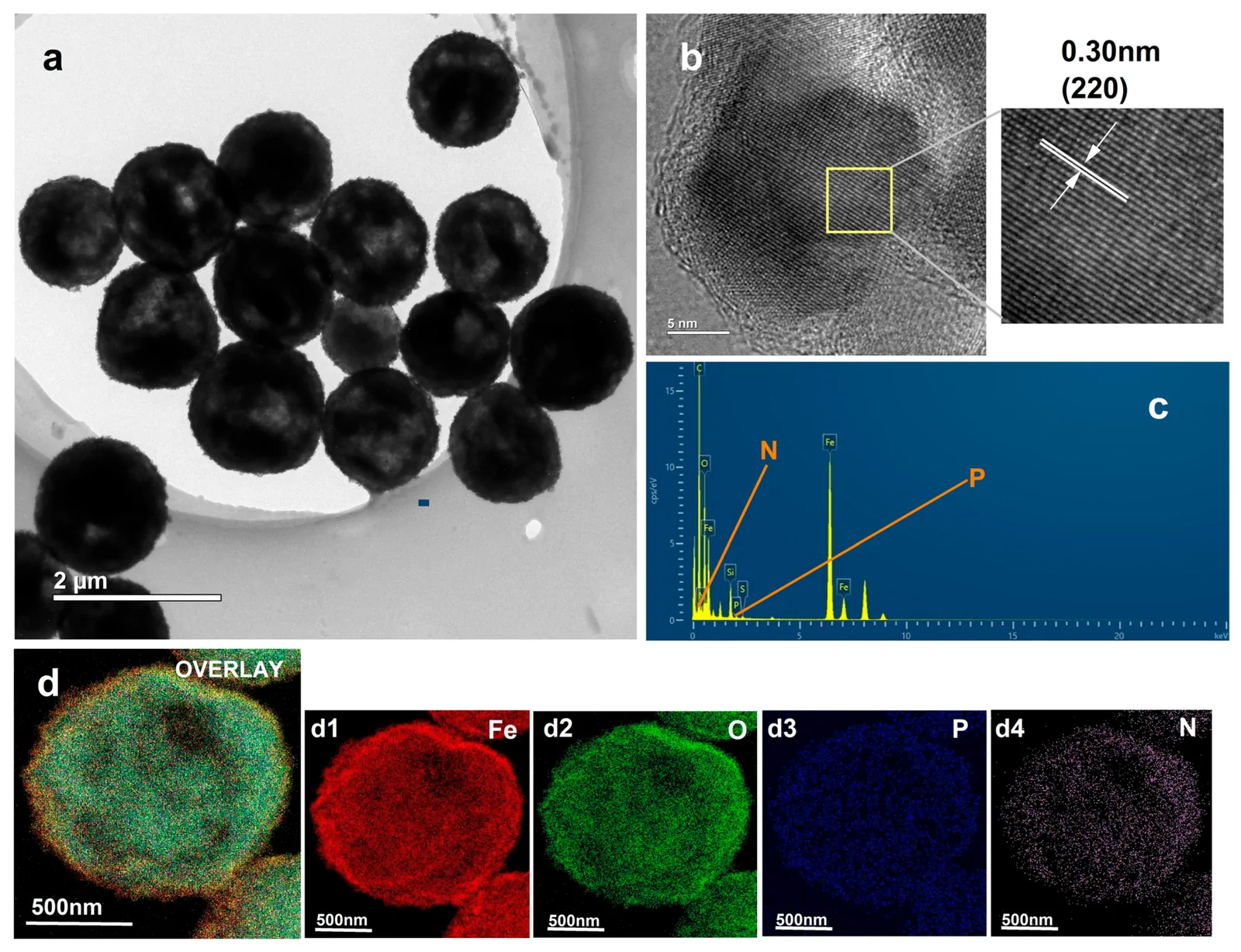
Morphology and structural characterization of the magnetic nanoparticles. (**a**) Low-magnification TEM image showing the general morphology and size distribution; (**b**) High-resolution TEM (HRTEM) image featuring the crystalline lattice fringe with a measured d-spacing of 0.30 nm corresponding to the (220) plane; (**c**) EDS spectrum demonstrating the elemental composition; and (**d**,**d1**–**d4**) EDS elemental mapping images visualizing the spatial distribution of Fe, O, P, and N elements over an individual nanoparticle.

High-resolution transmission electron microscopy (HRTEM) was performed to investigate the ultrastructure of the MNPs. As shown in Figure 3, the HRTEM image clearly reveals highly ordered crystalline lattices with an interplanar spacing of 0.30 nm, which is assignable to the (220) crystallographic plane of the Fe_3_O_4_ core (Figure 3b). Furthermore, a thin amorphous layer is clearly visible at the periphery of the crystalline core, which is attributed to the surface polymeric coating and the conjugated biological probes (streptavidin and DNA duplexes), validating successful surface functionalization. The general morphology and elemental composition of the MNPs were further characterized by TEM and EDS, as presented in Figure 3a,c,d,d1–d4.

**Figure 4:**
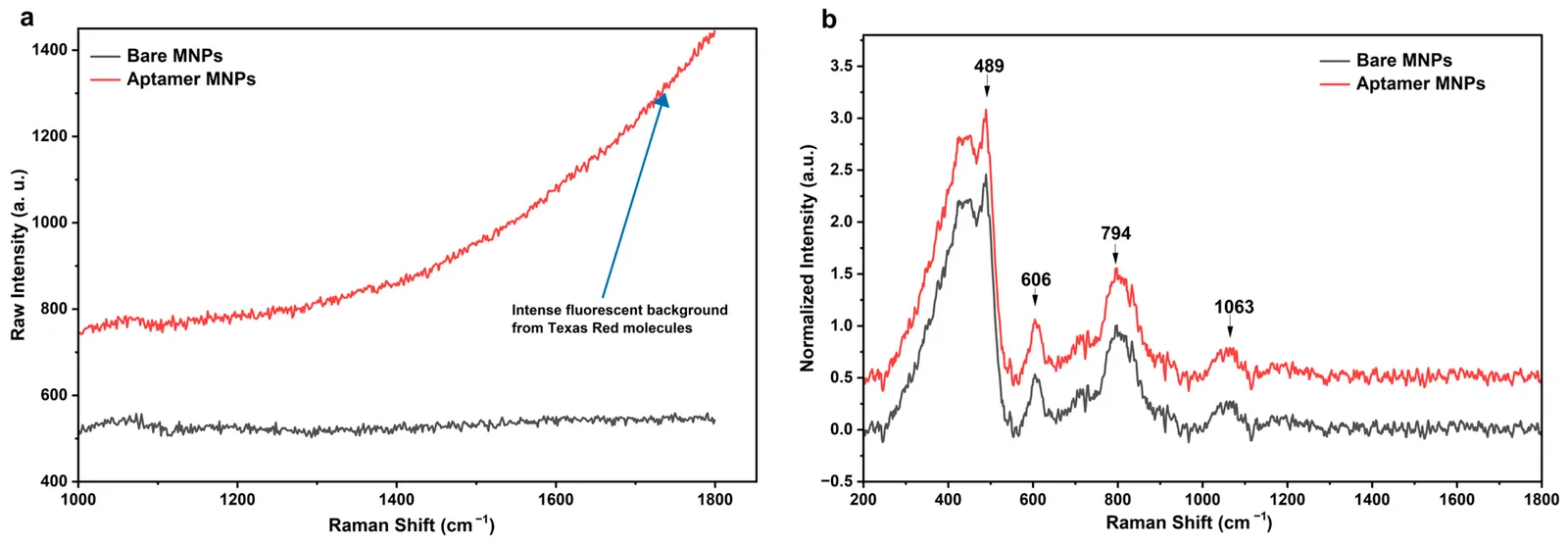
Raman spectroscopic evaluation of the surface functionalization process. (**a**) Raw Raman spectra of bare SA-MNPs (blank control) and Aptamer-MNPs within the high-wavenumber region, highlighting the intense fluorescence background arising primarily from the Texas Red molecules that appeared to be bound to the aptamer; (**b**) Baseline-corrected and normalized Raman spectra resolving the surface molecular coordination and skeletal protein fingerprints (489, 606, 794, and 1063 cm^−1^), suggesting that the nucleotide assembly was compatible with the underlying streptavidin layers and did not cause major disruption.

The functionalization process was evaluated via a complementary spectral strategy (Figure 4). As shown in Figure 4a, the raw Raman spectra of bare SA-MNPs (blank control) and Aptamer-MNPs within the high-wavenumber region were compared. Considered as an initial qualitative indication, the raw Raman spectrum of Aptamer-MNPs within the high-wavenumber region (Figure 4a) exhibited an abrupt, steep baseline uplift starting from 1400 cm^−1^ up to the acquisition limit of 1800 cm^−1^. This typical radiant slope appears to be primarily attributable to the dominant fluorescence emission background of the Texas Red molecules that seemed to be associated with the aptamer, which was absent in the bare SA-MNPs profile. To circumvent this crosstalk and resolve the surface molecular coordination, baseline correction and normalization to the internal reference peak at 794 cm^−1^ were systematically implemented (Figure 4b). The baseline-corrected and normalized Raman spectra showed that the principal streptavidin-associated spectral features at 489, 606, 794, and 1063 cm^−1^ remained detectable after probe assembly. Together with the elevated fluorescence background attributed to Texas Red, these observations are consistent with the association of the labeled oligonucleotide components with the MNP surface. However, Raman spectroscopy alone did not quantify probe loading, establish loading reproducibility, or independently verify the molecular integrity and binding activity of the immobilized aptamer [32].

To further quantify oligonucleotide functionalization and assess batch-to-batch reproducibility, fluorescence-based mass-balance analysis was performed using three independently prepared MNP probe batches. After cDNA immobilization and the corresponding washing steps, the cDNA-FAM immobilization efficiency ranged from 78.3% to 82.3%, with a mean value of 80.4 ± 2.0% and an inter-batch RSD of 2.5%. The corresponding batch-averaged loading amount was 964 ± 24 pmol mg^−1^ MNP.

For the Texas Red-labeled aptamer, the fluorescence mass balance included the post-hybridization supernatant and the three initial washing fractions collected before thermal equilibration and the subsequent five stringent washing steps. The apparent aptamer-retention efficiency after these initial washing steps ranged from 88.3% to 92.3%, with a mean value of 90.5 ± 2.0% and an inter-batch RSD of 2.2%. This corresponded to an apparent retained amount of 317 ± 7 pmol mg^−1^ MNP and an apparent aptamer-to-cDNA molar ratio of 0.328 ± 0.001 at this intermediate preparation stage.

These results support reproducible cDNA immobilization and reproducible initial aptamer association across the three independently prepared MNP batches. However, aptamer released during the subsequent thermal-equilibration and five stringent washing steps was not quantified. Therefore, the reported aptamer-retention value should not be interpreted as the final loading efficiency or final aptamer-to-cDNA stoichiometry of the ready-to-use probe. Functional reproducibility of the fully prepared probe batches was evaluated separately through their analytical responses, as reported in Table S3b. Detailed fluorescence mass-balance results are provided in Table S10a–c.

### 3.3. Optimization of Sensing Kinetics

#### 3.3.1. Feasibility Validation

**Figure 5:**
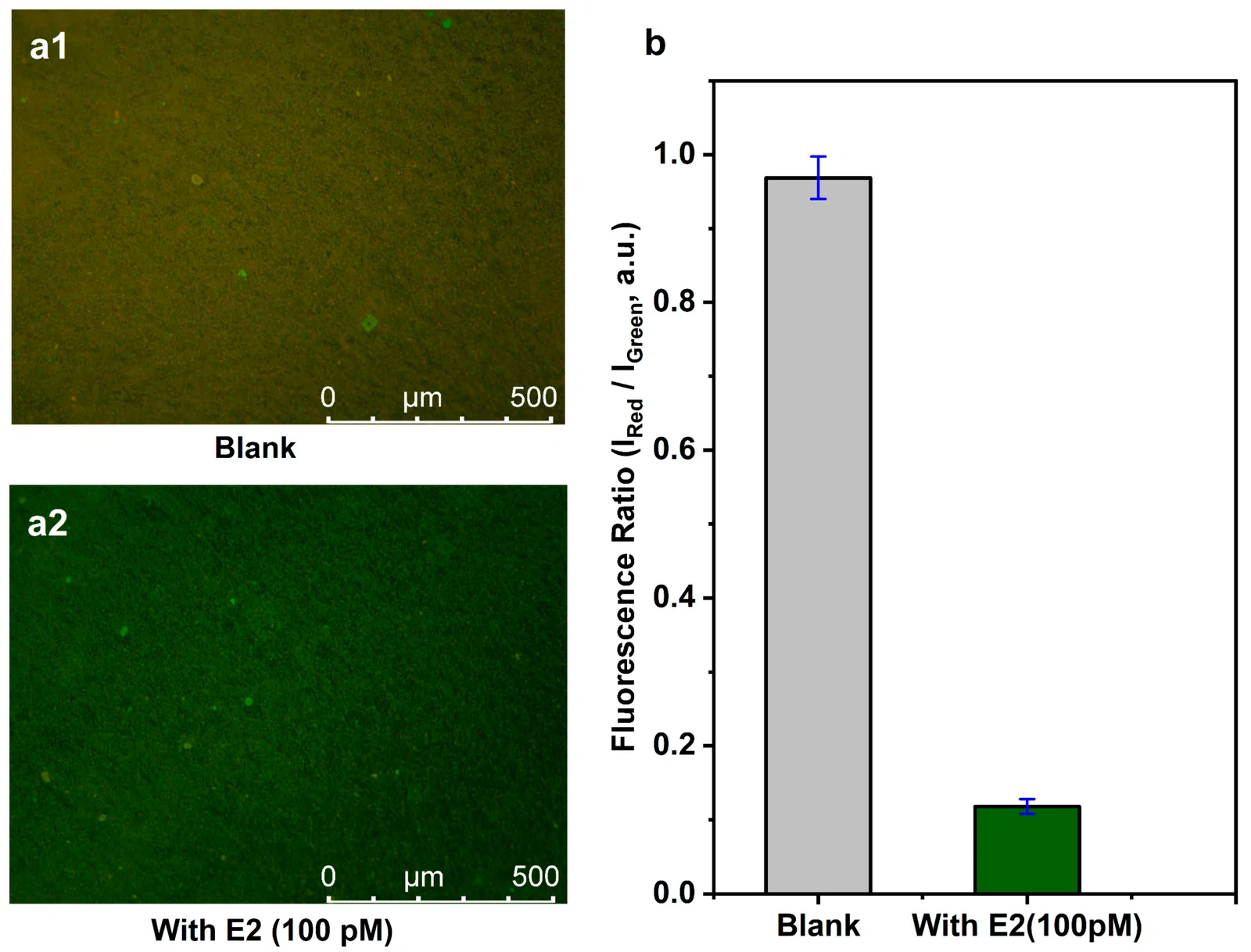
Feasibility of the ratiometric sensing strategy. (**a1**,**a2**) Pseudo-color fluorescence images of the AAO membrane after deposition of the sensing complexes in the absence (**a1**) and presence (**a2**) of 100 pM E2, showing a color transition from yellow-green to emerald green; (**b**) corresponding IRed/IGreen ratios confirming target-induced signal switching. Each data point represents one independently prepared sensing assay processed through magnetic separation, AAO loading, and fluorescence imaging. Data are presented as mean ± SD of three independent assays (*n* = 3).

To verify the practical utility of this ratiometric design, we examined the fluorescence response of the AAO-confined sensing layer upon addition of 100 pM E2. In the absence of target (Figure 5(a1)), the merged pseudo-color images showed a typical yellow-green emission, attributable to the combined contributions from both the FAM reference and the Texas Red reporter. Upon E2 challenge (Figure 5(a2)), a clear shift to emerald green was observed, implying efficient suppression of the Texas Red signal along with relative enhancement of the FAM channel. The corresponding intensity ratios (*I_Red_*/*I_Green_*) were compared in Figure 5b; a marked decrease was found for the E2-treated group relative to the blank control (*p* < 0.001). Collectively, these observations support the occurrence of target-induced strand displacement with sufficient sensitivity at low picomolar concentrations.

#### 3.3.2. Optimization of the Probe Concentration Ratio

**Figure 6:**
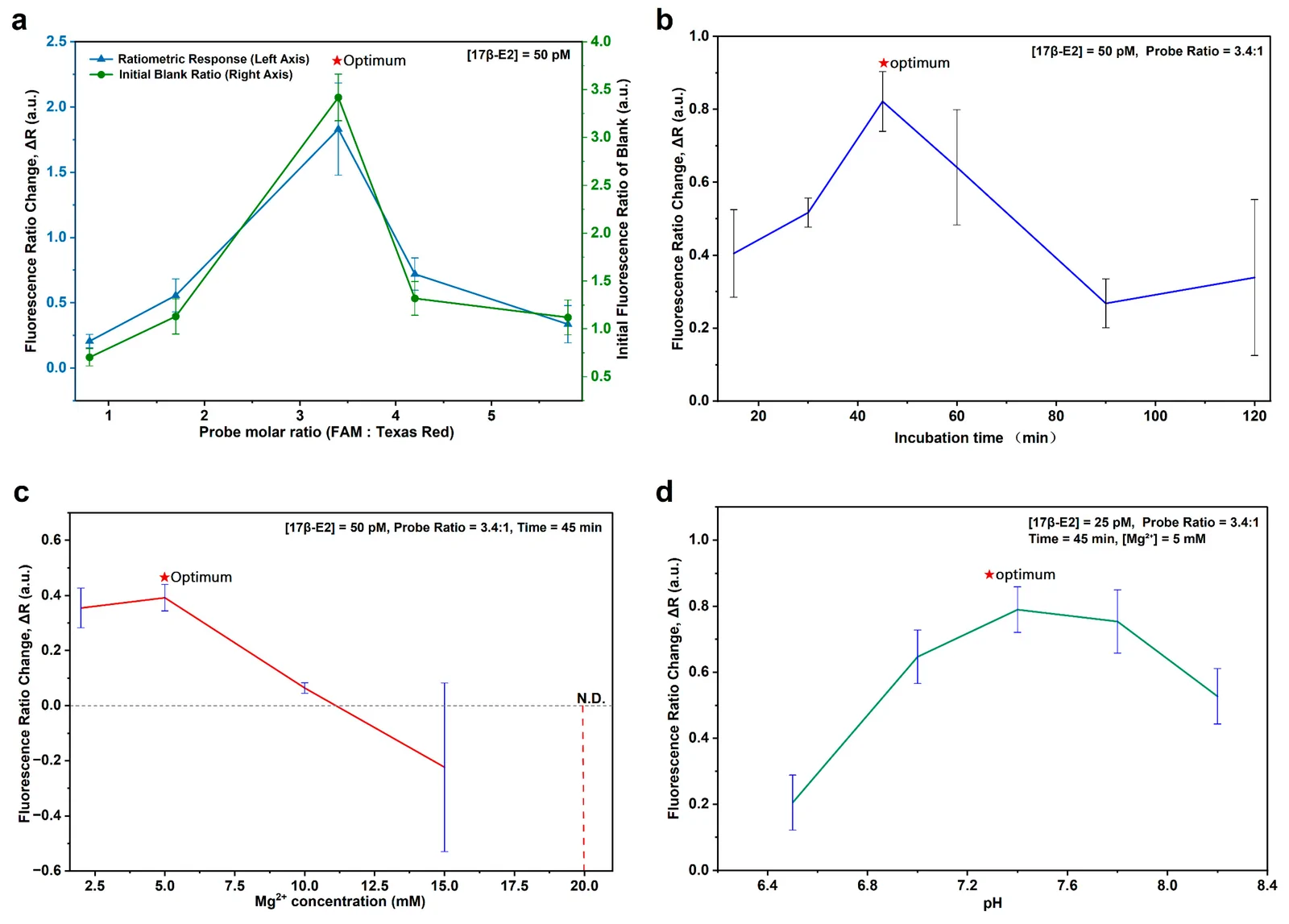
Systematic optimization of experimental parameters for the ratiometric fluorescence sensor. (**a**) Optimization of the molar ratio between the FAM-labeled and Texas Red-labeled aptamer probes; (**b**) effect of incubation time on the sensor response; (**c**) optimization of the Mg^2+^ concentration in the reaction system; and (**d**) effect of pH on the ratiometric response The 17β-estradiol (E2) concentration was maintained at 50 pM during the optimization experiments shown in panels (**a**–**c**) and at 25 pM during the pH optimization shown in panel (**d**). The net ratiometric response (ΔR) was calculated as ΔR = R_0_− R_E2_, where R_0_ and R_E2_ denote the fluorescence ratios in the absence and presence of E2, respectively. N.D. denotes ‘not detected’. Error bars represent the SD of three independently prepared exploratory assays per condition (*n* = 3).

The density and stoichiometry of signaling probes on the MNP surface directly affect the baseline fluorescence and, ultimately, the sensitivity. We therefore tested five molar ratios of FAM to Texas Red-labeled aptamer probes—0.8:1, 1.7:1, 3.4:1, 4.2:1, and 5.8:1—to see how this parameter influences the response (Figure 6a). The ratiometric signal increased progressively as the ratio went from 0.8:1 up to 3.4:1, where the maximum change was observed. This suggests that at this stoichiometry, the interfacial arrangement is optimal—steric hindrance is kept reasonably low while target-induced conformational switching remains efficient. Beyond that point, however, the performance dropped noticeably at 4.2:1 and 5.8:1. We attribute this decline to overcrowding of the probes on the nanoparticle surface, which likely causes self-quenching or restricts the conformational freedom required for effective strand displacement. Based on these results, a FAM-to-aptamer ratio of 3.4:1 was adopted for all subsequent experiments.

#### 3.3.3. Incubation Time Kinetics

The ratiometric response increased up to approximately 45 min and then gradually decreased through 120 min. Therefore, 45 min was selected as the working incubation time based on the maximum observed response rather than the attainment of a stable plateau. The subsequent decrease may reflect time-dependent interfacial redistribution, probe rehybridization, altered bead dispersion, or changes in the aptamer–target/aptamer–cDNA equilibrium; however, these possibilities were not independently investigated.

#### 3.3.4. Optimization of Mg^2+^ Concentration

The effect of Mg^2+^ concentration on the sensor response was examined over the range of 2–20 mM, with the E2 concentration fixed at 50 pM (Figure 6c). The ratiometric signal increased as the Mg^2+^ concentration increased to 5 mM, reached its maximum at this concentration, and subsequently decreased at higher Mg^2+^ concentrations. This result suggests a concentration-dependent dual effect of Mg^2+^ on the sensing process. At an appropriate concentration, Mg^2+^ may promote the formation of the target-binding conformation of the aptamer while maintaining suitable aptamer–cDNA hybridization, thereby facilitating efficient E2 recognition and target-induced strand displacement.

When the Mg^2+^ concentration exceeded 10 mM, however, the ratiometric response decreased markedly. Excessive Mg^2+^ may over-stabilize the aptamer–cDNA duplex, thereby hindering E2-induced aptamer dissociation and strand displacement. High Mg^2+^ concentrations may also stabilize compact or alternative aptamer conformations in which the E2-binding region is less accessible. In addition, Mg^2+^-mediated interactions between the negatively charged DNA phosphate backbone and the MNP or AAO surfaces may alter probe accessibility, magnetic recovery, and the spatial distribution of the fluorescent complexes within the nanochannels.

At 15 mM Mg^2+^, no positive E2-induced ratiometric response was observed, while at 20 mM Mg^2+^, the target-induced response could no longer be distinguished from the background and was therefore recorded as not detected (N.D.). This loss of response may result from the combined effects of impaired strand displacement, altered aptamer conformation, and Mg^2+^-mediated probe–surface interactions. Moreover, because a phosphate-containing buffer was used, the possible formation of Mg–phosphate-associated species or microprecipitates at elevated Mg^2+^ concentrations cannot be excluded; these species could contribute to probe sequestration, heterogeneous AAO loading, or optical scattering. Based on the maximum ratiometric response, 5 mM Mg^2+^ was selected as the working concentration for subsequent experiments.

#### 3.3.5. Optimization of the Reaction pH

The pH of the reaction medium can influence aptamer folding, aptamer–cDNA hybridization, target recognition, and the fluorescence behavior of the labeled probes. We therefore evaluated the sensor response over a pH range from 6.5 to 8.2, with the E2 concentration fixed at 25 pM (Figure 6d). The net ratiometric response increased markedly from 0.205 at pH 6.5 to 0.647 at pH 7.0 and reached its maximum value of 0.790 at pH 7.4. When the pH was further increased to 7.8, the response decreased slightly to 0.754, corresponding to 95.4% of the maximum response. A more pronounced decline was observed at pH 8.2, where the response fell to 0.527.

The relatively weak response under mildly acidic conditions may arise from changes in the protonation state of the nucleobases and fluorophores, which can affect aptamer folding, duplex stability, and fluorescence output. In contrast, moderately alkaline conditions may partially disturb the noncovalent interactions required for stable aptamer–target recognition or alter the equilibrium between the aptamer and its complementary strand. The response remained comparatively high between pH 7.0 and 7.8, indicating that the sensing system has reasonable tolerance to small pH variations around neutrality. Nevertheless, pH 7.4 produced the highest mean response and showed better repeatability than pH 7.8. It also corresponds to the nominal pH of the commercial 1× PBS-T buffer used throughout the assay. Accordingly, pH 7.4 was selected as the working condition for all subsequent experiments.

### 3.4. Analytical Performance and Matrix Effects

**Figure 7:**
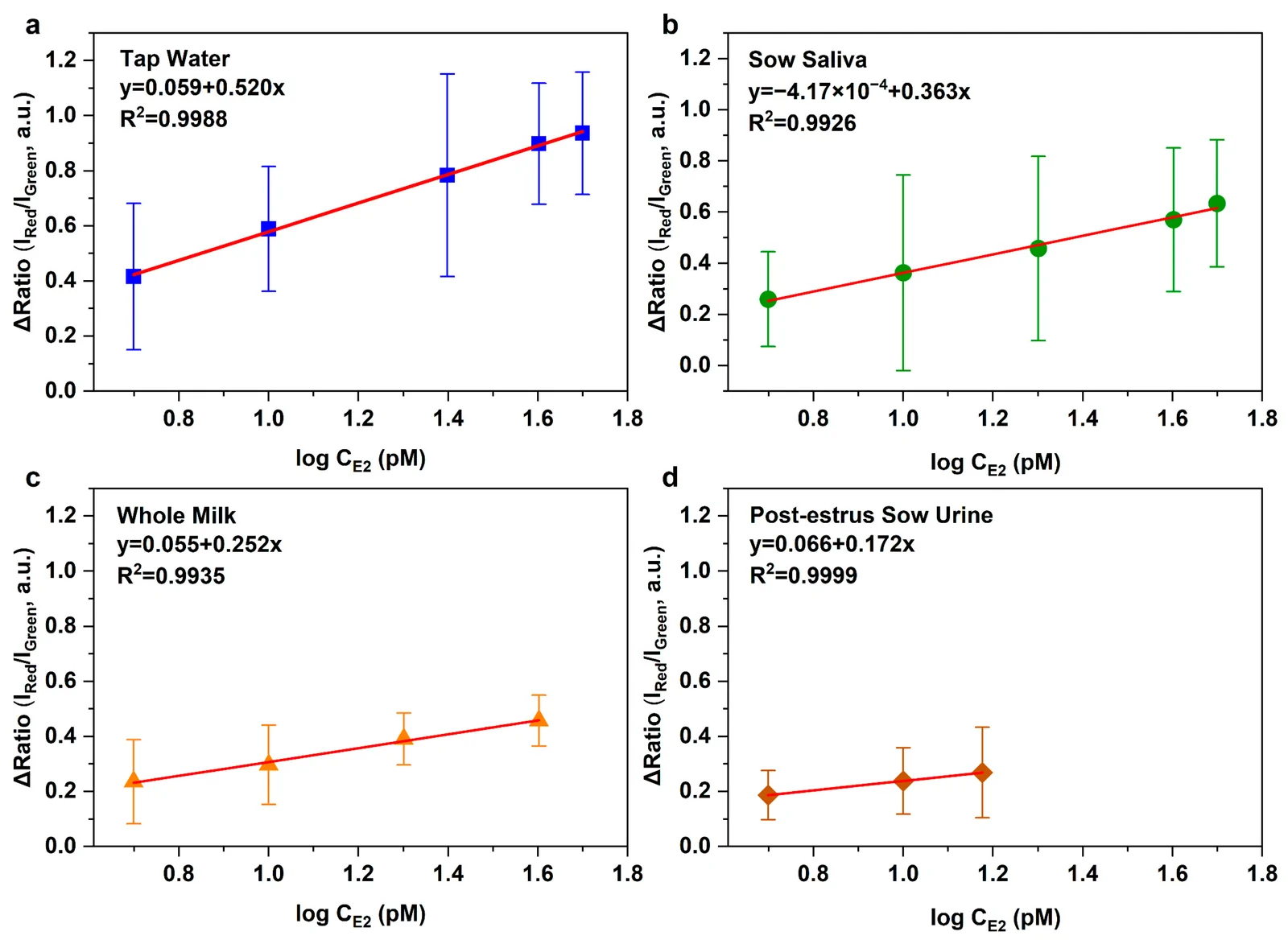
Matrix-specific calibration plots of the ratiometric fluorescent aptasensor for 17β-estradiol (17β-E2) determination. The *x*-axis represents the logarithmically transformed E2 concentration, *x* = log_10_(*C*_E2_/pM). The concentration levels used were (**a**) 5, 10, 25, 40, and 50 pM in tap water; (**b**) 5, 10, 20, 40, and 50 pM in sow saliva; (**c**) 5, 10, 20, and 40 pM in whole milk; and (**d**) 5, 10, and 15 pM in post-estrus sow urine. The regression equations and coefficients of determination (*R*^2^) are shown in the corresponding panels. Data are presented as the mean ± SD of five independently prepared assays at each concentration level (*n* = 5). In panels (**a**–**d**), the markers with distinct shapes and colors (blue squares, green circles, orange triangles, and red diamonds) denote the mean values in tap water, sow saliva, whole milk, and post-estrus sow urine, respectively. The vertical error bars represent the corresponding SDs, and the solid red lines illustrate the linear regression fits for each matrix. The LOD in tap water was determined using ten independently prepared blank tap-water assays (*n* = 10) and the corresponding tap-water calibration equation, yielding an LOD of 3.62 pM. Because only three calibration levels were available for urine, the urine model should be interpreted as a preliminary limited-range calibration. Detailed concentration levels and regression statistics are provided in Table S9.

To evaluate the analytical response of the ratiometric aptasensor and the influence of sample matrices, matrix-specific calibration experiments were conducted in tap water, saliva, whole milk, and post-estrus sow urine. Each concentration level was evaluated using five independently prepared assays (*n* = 5), and the results are presented as the mean ± SD in Figure 7. Linear relationships were established between the baseline-corrected ratiometric response (ΔR) and the logarithm of the 17β-E2 concentration (logC_E2_) over 5.0–50.0 pM in tap water and saliva, 5.0–40.0 pM in whole milk, and 5.0–15.0 pM in post-estrus sow urine. The corresponding regression equations and coefficients of determination (R^2^) are shown in Figure 7a–d, whereas the standard errors and 95% confidence intervals of the regression slopes are summarized in Table S9.

The slope standard errors and 95% confidence intervals provide additional information on the uncertainty of the fitted slope parameters beyond the R^2^ values. However, residual-versus-fitted plots and formal tests of homoscedasticity were not performed in the present study. Consequently, random residual distribution and constant residual variance were not independently established, and the reported regression statistics should not be interpreted as a complete assessment of calibration-model adequacy.

The LOD in tap water was recalculated using ten independently prepared blank tap-water assays. The response threshold was defined as the mean blank response plus three standard deviations, and the corresponding E2 concentration was obtained by back-calculation through the logarithmic tap-water calibration equation. The resulting LOD was 3.62 pM. Concentration levels below this value were therefore excluded from the final quantitative calibration models. The final matrix-specific calibration ranges were 5.0–50.0 pM for tap water and saliva, 5.0–40.0 pM for whole milk, and 5.0–15.0 pM for post-estrus sow urine.

Although satisfactory linear relationships were obtained within the validated range of each matrix, the calibration slopes differed substantially. The slopes were 0.520 for tap water, 0.363 for saliva, 0.252 for whole milk, and 0.172 for post-estrus sow urine. Relative to tap water, the reduced slopes in the biological matrices indicate lower analytical sensitivity and demonstrate that residual matrix-dependent effects remained after magnetic separation, ratiometric correction, and AAO nanochannel confinement. Therefore, the sensing system should not be considered matrix independent, and the corresponding matrix-specific calibration equations were used for quantitative analysis.

The matrix-specific baseline ratios also differed markedly. The mean R0values were 3.663 ± 0.089 in tap water, 2.598 ± 0.114 in sow saliva, 1.297 ± 0.096 in whole milk, and 0.933 ± 0.056 in post-estrus sow urine, with detailed values provided in Table S9. These results indicate that different sample matrices influenced both the initial fluorescence-ratio baseline before target addition and the incremental calibration sensitivity. Therefore, the reduced calibration slopes observed in biological matrices cannot be attributed solely to reduced target accessibility or suppressed aptamer–target binding. Matrix-dependent optical effects, nonspecific interactions, probe redistribution, and variations in target-recognition efficiency may all contribute to the observed matrix dependence. The present experiments were designed to characterize the overall matrix effect and did not independently deconvolute the contribution of each individual mechanism.

In addition to these differences in calibration sensitivity, the interpretation of the biological-matrix calibration curves requires consideration of the matrix-specific baseline correction. For each biological matrix, the unspiked pretreated sample was used to establish the baseline response. This correction accounted for the initial fluorescence response arising from the matrix and from any endogenous target present in the unspiked aliquot. However, baseline correction should not be interpreted as the physical removal or complete elimination of endogenous E2 or other matrix components. Consequently, the biological-matrix calibration models describe the incremental response to added E2 above the corresponding sample background rather than the absolute total E2 concentration in the original sample.

This distinction was particularly relevant for post-estrus sow urine. ELISA analysis indicated that the undiluted pooled urine contained approximately 360–380 pM E2, corresponding to approximately 3.6–3.8 pM after the 100-fold dilution used for aptasensor analysis. This endogenous E2 was present in both the unspiked and spiked urine aliquots and was therefore incorporated into the matrix-specific baseline response. Accordingly, the urine regression model describes the response to 5–15 pM of added E2 above the diluted urine background, rather than the absolute total E2 concentration in the original urine sample.

The lower sensitivity observed in whole milk may be associated with its high protein, lipid, and colloidal content. Residual macromolecules may restrict target diffusion, adsorb onto the magnetic probe or AAO surface, and introduce steric hindrance around the aptamer recognition interface, thereby reducing the effective interaction between 17β-E2 and the aptamer [33,34]. During preliminary evaluation, the response at 50 pM showed deviation from the linear trend and was therefore excluded from the final milk calibration model, resulting in a validated calibration range of 5.0–40.0 pM. This finding further indicates that the applicable quantitative range depends on the composition of the sample matrix.

Post-estrus sow urine was diluted 100-fold before analysis. This pretreatment reduced the concentrations of urea, inorganic salts, and other urinary components that could affect aptamer conformation, target recognition, or fluorescence readout. It also reduced the endogenous E2 background and maintained the analytical response within the measurable range of the assay. In addition, the urine calibration was established using only three concentration levels over 5.0–15.0 pM and should therefore be regarded as a preliminary limited-range model. Its high *R*^2^ value and narrow slope confidence interval should not be interpreted as evidence of complete model adequacy because the residual pattern and homoscedasticity were not formally evaluated. Validation using additional independently prepared concentration levels and independent urine samples will be required to support a more comprehensive quantitative model.

Collectively, the observed tolerance to complex matrices resulted from the combined effects of sample pretreatment, magnetic separation, ratiometric correction, and AAO-assisted solid-state fluorescence collection. Matrix-specific pretreatment reduced proteins, suspended materials, salts, and other endogenous components before analysis. Magnetic separation and washing further removed a proportion of the soluble or weakly associated matrix constituents. The dual-channel ratiometric signal partially compensated for common variations in probe loading, fluorescence excitation, and image acquisition, while AAO nanochannel confinement reduced nonuniform lateral redistribution of the magnetic sensing complexes and improved the spatial reproducibility of the solid-state fluorescence signal. Nevertheless, the substantially different calibration slopes obtained for tap water, saliva, whole milk, and urine demonstrate that these procedures reduced, but did not eliminate, matrix-dependent interference. The use of a tap-water calibration equation for biological samples would therefore introduce systematic quantification bias. Matrix-matched calibration was consequently applied to account for differences in analytical sensitivity among the investigated matrices.

To benchmark both the analytical performance and the practical implementation of the developed platform, representative recent ratiometric aptasensors for 17β-estradiol detection were compared in Table 2. For clarity, the comparison was divided into two complementary sections. Table 2a summarizes the analytical characteristics of the reported platforms, including linear range, limit of detection, real-sample application, and readout format, whereas Table 2b compares practical and operational factors, including total analysis time, washing requirements, reported stability, estimated consumable cost, preparation complexity, reusability, and instrumental complexity. Because several operational parameters were not consistently reported in the original studies, NR was used when the corresponding information could not be reliably obtained, and detailed information supporting the qualitative classifications is provided in Table S8.

The compared platforms achieve ratiometric E2 detection through different analytical strategies, including homogeneous FRET or LRET, paper-based smartphone imaging, dual electrochemical referencing, nucleic-acid amplification, and enzyme/nanozyme cascade amplification. Several of these methods provide broader calibration ranges, lower reported LODs, simpler homogeneous measurements, or more portable signal acquisition than the present system. Therefore, analytical sensitivity alone does not establish the overall superiority of any single platform.

The distinctive feature of the present platform is the sequential integration of three functions within one sensing workflow. Magnetic separation and washing reduce soluble matrix-associated components before fluorescence measurement; the dual-color signal provides internal ratiometric correction; and the AAO membrane spatially localizes the remaining particle-associated sensing complexes during solid-state fluorescence collection. Compared with conventional solution-phase ratiometric assays, this architecture adds control over the spatial distribution of the deposited sensing complexes and mitigates coffee-ring-associated fluorescence heterogeneity. However, these features are accompanied by operational trade-offs, including a 45-min recognition step, two magnetic washing steps, overnight batch-probe preparation, manual AAO loading, fluorescence-microscope imaging, and image analysis. Reuse was not demonstrated, and matrix-specific calibration remained necessary. Thus, the proposed platform should be viewed as a complementary solid-state sensing architecture with specific advantages in magnetic matrix cleanup and spatial fluorescence localization, rather than as uniformly superior to previously reported ratiometric E2 aptasensors.

The comparison indicates that lower detection limits do not necessarily correspond to simpler analytical workflows. The present method provides magnetic isolation and localized solid-state ratiometric imaging, but its overnight batch-probe preparation, manual post-recognition handling, microscope-based readout, and lack of demonstrated reusability remain important limitations. Further simplification and integration of these procedures will be required before routine on-site implementation.

### 3.5. Selectivity and Cross-Reactivity Evaluation and Molecular Docking Analysis

**Figure 8:**
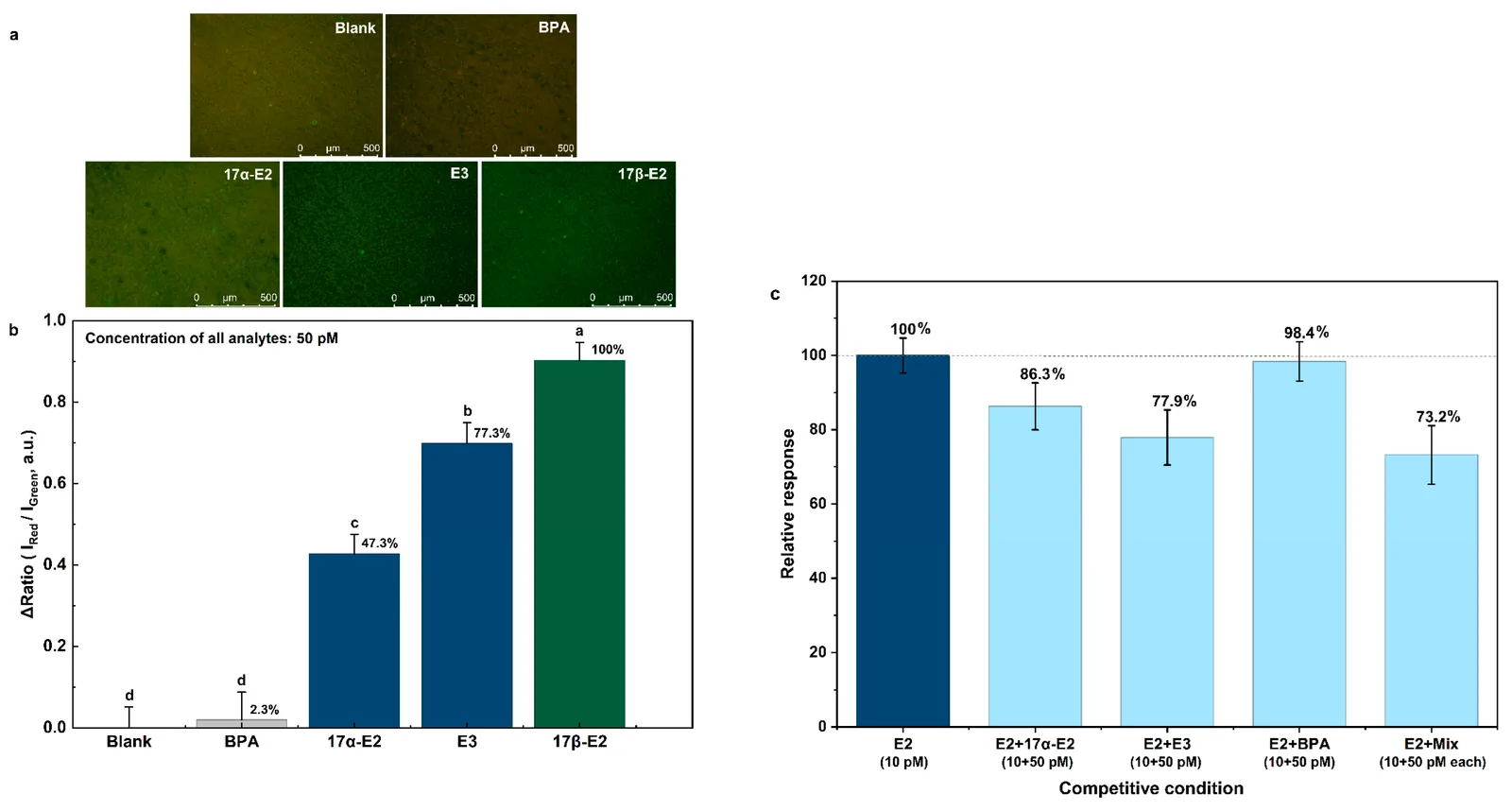
Individual cross-reactivity and competitive-interference assessment of the ratiometric aptasensor under analytically relevant concentration conditions. (**a**) Representative merged dual-color fluorescence images of the blank control, bisphenol A (BPA), 17α-estradiol (17α-E2), estriol (E3), and 17β-estradiol (17β-E2) groups. All analytes were individually tested at 50 pM. All fluorescence images were acquired and displayed using identical excitation intensity, exposure time, detector gain, and image-processing settings. Scale bar = 100 μm. (**b**) Corresponding quantitative ΔR responses. ΔR was calculated as ΔR = R_blank − R_sample, where R = I_Red/I_Green. Bars represent the mean ± SD of five independently prepared assays (*n* = 5). The percentages shown above the bars represent relative cross-reactivity normalized to the response of 17β-E2, which was defined as 100%. Different lowercase letters indicate statistically significant differences according to one-way ANOVA followed by Tukey’s multiple-comparison test (*p* < 0.05). (**c**) Quantitative competitive-interference responses under excess-interferent conditions. A fixed concentration of 10 pM 17β-E2 was analyzed alone or in the presence of a fivefold molar excess (50 pM) of 17α-E2, E3, BPA, or a mixture of all three interferents. Relative responses were calculated by normalizing the E2-only response to 100%. The dashed horizontal line in (**c**) represents the 100% relative response baseline defined by the 17β-E2-only control. Data represent the mean ± SD of five independently prepared assays (*n* = 5).

To obtain a selectivity assessment consistent with the analytical working range of the aptasensor, the available potential interferents were individually evaluated at 50 pM. Representative merged dual-color fluorescence images of the blank control, BPA, 17α-E2, E3, and 17β-E2 groups are shown in Figure 8a, while the corresponding quantitative ΔR responses are presented in Figure 8b. All images were acquired and processed using identical optical and display settings. Figure 8c separately presents the quantitative competitive-interference responses obtained when 10 pM 17β-E2 was analyzed in the presence of fivefold excess interferents.

At an equal concentration of 50 pM, the mean ΔR values obtained for 17β-E2, 17α-E2, E3, and BPA were 0.902 ± 0.044, 0.427 ± 0.048, 0.698 ± 0.051, and 0.020 ± 0.068, respectively, compared with 0.000 ± 0.052 for the blank control. When the response toward 17β-E2 was defined as 100%, the relative cross-reactivity values of 17α-E2, E3, and BPA were 47.3%, 77.3%, and 2.3%, respectively.

One-way ANOVA followed by Tukey’s multiple-comparison test showed that the response toward 17β-E2 was significantly higher than those toward E3 and 17α-E2. E3 also produced a significantly higher response than 17α-E2, whereas the BPA response was not significantly different from that of the blank control. The negligible response toward BPA indicates limited response toward this structurally dissimilar non-steroidal compound.

In contrast, the appreciable responses toward E3 and 17α-E2 confirm substantial cross-reactivity with closely related estrogen analogs. E3 and 17α-E2 share the same cyclopentanophenanthrene steroid framework as 17β-E2 and differ mainly in the number, position, or stereochemical orientation of their hydroxyl groups. These structural similarities may contribute to partial recognition by the E2 aptamer and consequently trigger reporter-strand displacement.

Therefore, the present aptasensor should be described as exhibiting a preferential response toward 17β-E2 among the tested analytes rather than absolute or exclusive specificity for 17β-E2. To further evaluate the quantitative response under more challenging competitive conditions, 10 pM 17β-E2 was analyzed alone or in the presence of a fivefold molar excess of the selected interferents, as summarized quantitatively in Figure 8c. When 10 pM 17β-E2 was analyzed alone, the relative response was defined as 100%. The addition of 50 pM 17α-E2, E3, or BPA resulted in relative responses of 86.3%, 77.9%, and 98.4%, respectively. In the presence of all three interferents simultaneously, the response remained at 73.2%. These results demonstrate that excess structurally related estrogens can partially influence the analytical response, particularly E3 and 17α-E2, while the aptasensor maintains a preferential response toward 17β-E2 under the tested competitive conditions. Magnetic separation and AAO nanochannel confinement reduce matrix-derived interference and improve the uniformity of the fluorescence readout; however, they do not alter the intrinsic molecular-recognition characteristics of the aptamer and therefore cannot eliminate cross-reactivity with structurally related estrogens. Because E3, 17α-E2, and other estrogen metabolites may coexist with 17β-E2 in urine and saliva, the aptasensor response in biological samples may contain contributions from these cross-reactive compounds. Matrix-matched calibration can compensate for matrix-dependent signal behavior but cannot provide compound-specific discrimination. Confirmatory chromatographic analysis or an aptamer with improved molecular selectivity would therefore be required for definitive 17β-E2 quantification in samples containing multiple natural estrogens.

It should be noted that the present competitive evaluation was limited to the selected interferents and concentration conditions investigated in this study. Although excess-interferent experiments partially addressed multi-analyte coexistence, a broader panel of steroid hormones, metabolites, and representative protein-associated interferents should be included in future validation studies to further characterize anti-interference performance in complex biological matrices.

**Figure 9:**
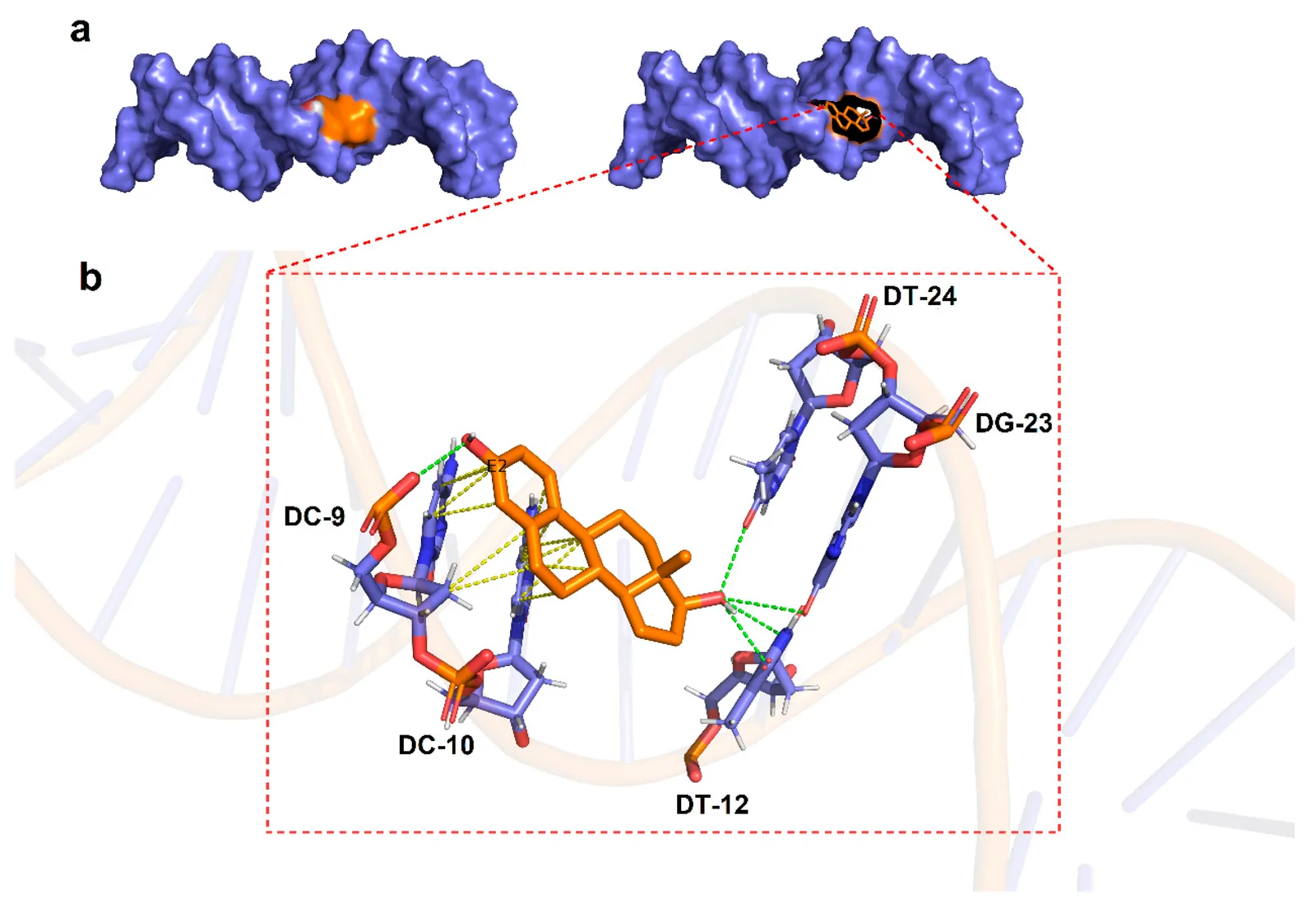
Predicted molecular docking pose of E2 with the aptamer. (**a**) Surface representation of the top-ranked computational pose, showing E2 positioned within a putative groove of the modeled aptamer structure. (**b**) Two-dimensional representation of the predicted binding pose. The dashed lines indicate putative polar and hydrophobic/van der Waals contacts identified from the computational model. The docking results represent a structural hypothesis and have not been independently validated by residue-level or biophysical binding experiments.

To investigate the structural basis of E2 recognition by the aptamer, molecular docking simulations were performed. The top-ranked docking pose yielded an AutoDock Vina score of −7.171 kcal·mol^−1^ (Figure 9a). As shown in the docking pose, In the predicted pose, E2 was positioned within a putative binding region involving DC9, DC10, DT12, DG23, and DT24 (Figure 9b).

Analysis of the intermolecular interactions revealed that DC9, DT12, DG23, and DT24 were predicted to participate in possible polar contacts with E2, while DC9 and DC10 contribute to hydrophobic/van der Waals interactions. Notably, DC9 was positioned near both putative polar and hydrophobic contacts in the computational pose and may contribute to ligand stabilization.

The conformational rearrangement of the aptamer upon target binding has been documented in previous work. Wang et al. reported that E2 binding induces a conformational change in the aptamer’s binding pocket, resulting in a 200% fluorescence enhancement of SYBR Green I—the highest reported enhancement for a label-free E2 aptasensor to date [41]. This previous observation is broadly compatible with the target-responsive behavior of the present aptasensor. However, it does not independently validate the specific binding pose or the residue-level contacts predicted by the docking model.

This predicted binding pose provides one possible structural hypothesis for the observed cross-reactivity profiles. Because E3 and 17α-E2 possess highly similar steroidal frameworks, these analogs may retain partially conserved hydrophobic and polar interactions within the predicted aptamer-binding region. Such partially shared interactions are consistent with the experimentally observed cross-reactivity of 77.3% for E3 and 47.3% for 17α-E2. Therefore, the docking results support a possible E2-binding mode but do not demonstrate that the current aptamer can unequivocally discriminate 17β-E2 from closely related natural estrogens.

### 3.6. Real Sample Analysis

#### 3.6.1. Matrix-Specific Spike-Recovery Evaluation

**Figure 10:**
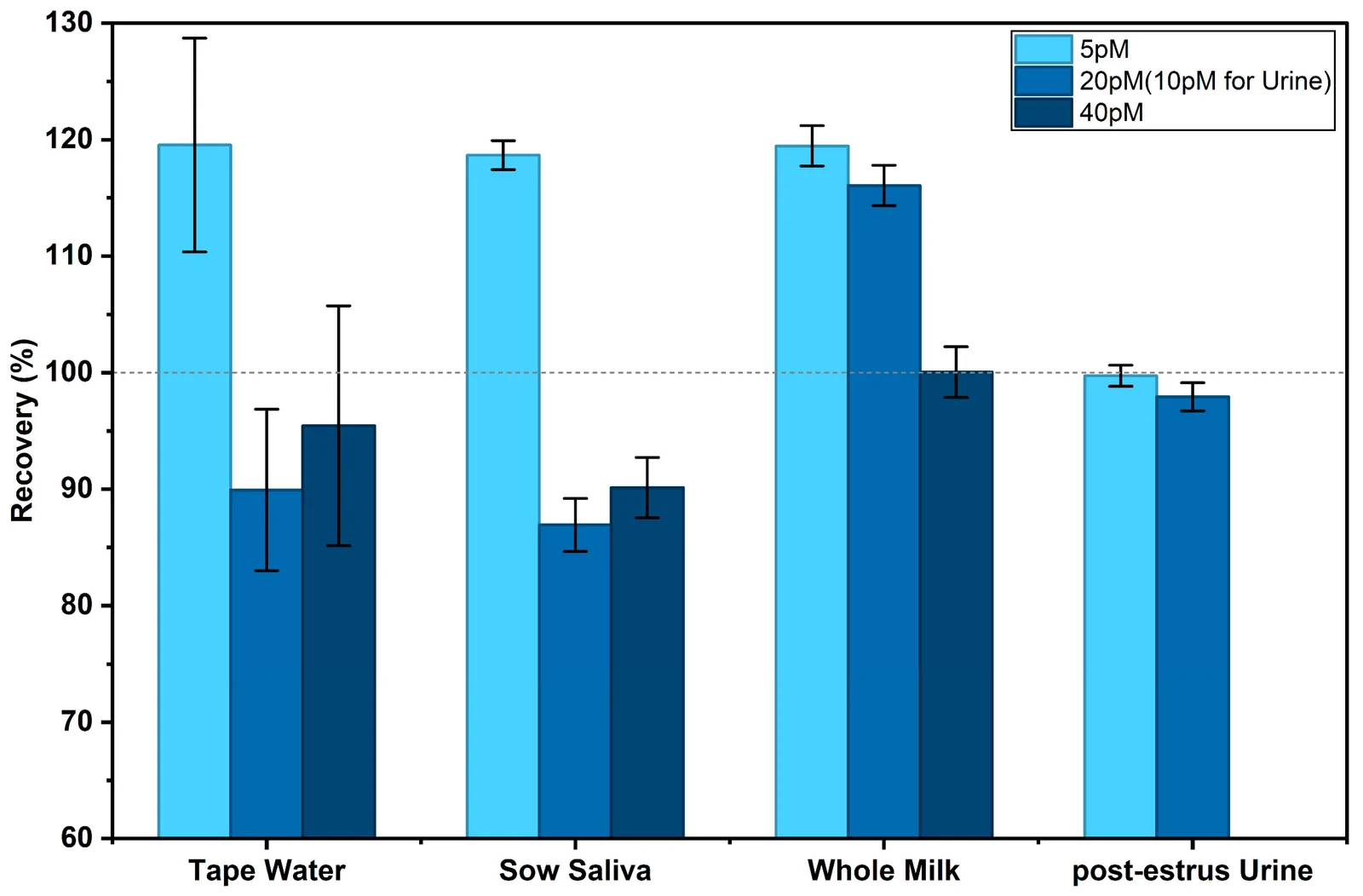
Matrix-matched spike-recovery evaluation of the ratiometric aptasensor in tap water, sow saliva, whole milk, and 100-fold diluted pooled post-estrus sow urine. Tap water, saliva, and milk were fortified at added E2 concentrations of 5, 20, and 40 pM, whereas diluted urine was fortified at 5 and 10 pM. The urine levels were selected within the experimentally evaluated added-concentration range of the urine calibration model (5–15 pM); urine samples fortified at 20 and 40 pM were therefore not evaluated. Data are presented as the mean ± SD of three independently prepared spiked samples at each concentration level (*n* = 3). Each replicate underwent the complete sample-preparation and sensing workflow independently. The overall recoveries ranged from 86.92% to 119.54%. The dashed horizontal line at 100% represents the ideal baseline for complete (100%) recovery of the spiked target.

Matrix-matched spike-recovery experiments were conducted to evaluate the quantitative performance of the aptasensor in the four investigated matrices (Figure 10). In tap water, the recoveries were 119.54%, 89.93%, and 95.45% at added E2 concentrations of 5, 20, and 40 pM, respectively. The corresponding recoveries in sow saliva were 118.67%, 86.92%, and 90.13%, whereas those in whole milk were 119.47%, 116.07%, and 100.05%. In the 100-fold diluted post-estrus sow urine, recoveries of 99.73% and 97.91% were obtained at added concentrations of 5 and 10 pM, respectively. The overall recovery range across the four matrices was therefore 86.92–119.54%.

The urine results were close to the nominal added concentrations within the limited urine calibration range. In tap water, saliva, and milk, the recoveries at 5 pM were close to the upper end of the overall recovery interval, indicating a modest positive bias near the lower ends of the corresponding calibration ranges. This tendency may reflect the greater influence of baseline-response variation, residual matrix effects, and inverse-calculation uncertainty at low concentrations. Some matrix-dependent underestimation was also observed in saliva at 20 and 40 pM. Overall, the results support preliminary matrix-matched quantitative feasibility, while also demonstrating that analytical bias varied according to matrix type and concentration level.

To further characterize the endogenous E2 background in the urine matrix, the pooled post-estrus sow urine used in the recovery study was analyzed using a commercial enzyme-linked immunosorbent assay (ELISA) kit. The undiluted urine pool contained approximately 360–380 pM E2. After the 100-fold dilution applied before aptasensor analysis, the estimated endogenous E2 concentration in the diluted urine matrix was approximately 3.6–3.8 pM. This value was numerically close to, but slightly below, the lowest added concentration of 5 pM used to construct the urine calibration model.

The urine calibration model was established using added E2 concentrations of 5, 10, and 15 pM, with the unspiked diluted urine serving as the matrix-specific baseline. Spike-recovery experiments were conducted at added concentrations of 5 and 10 pM, both of which were within the experimentally evaluated calibration range. Because recovery was not evaluated at 15 pM or at concentrations above the calibration range, the recovery conclusions should be limited to the two tested addition levels.

Because only three added-concentration levels were used and the diluted urine retained an endogenous E2 background of approximately 3.6–3.8 pM, the urine calibration should be regarded as a preliminary matrix-matched incremental calibration. It describes the response to added E2 above the corresponding urine background and cannot, by itself, provide absolute quantification of the total endogenous E2 concentration in an unknown urine sample. Further validation using standard addition, an independently characterized E2-free or low-E2 urine matrix, or comparison with a validated matrix-specific reference method would therefore be required for absolute endogenous E2 determination.

A previous FRET-based aptasensor study also applied sample dilution before urinary E2 determination [15], indicating that dilution can lower the concentrations of endogenous E2 and other matrix constituents entering the assay and thereby attenuate urine-associated matrix effects. However, the ELISA measurement in the present study was performed only on the pooled urine batch used in these experiments. Therefore, the measured endogenous E2 concentration should not be generalized to all post-estrus sow urine samples, because urinary E2 levels may vary among animals, sampling times, and physiological stages.

#### 3.6.2. Comparison with ELISA

To provide an orthogonal comparison of the aptasensor results in biological matrices, spiked urine and saliva samples were analyzed in parallel using the aptasensor and a commercial estradiol ELISA kit. The urine used in this comparison was collected from non-estrous sows and was obtained from a separate sample batch from the pooled post-estrus sow urine used for the matrix-specific calibration and spike-recovery experiments described in Section 3.6.1.

Urine and saliva samples were fortified with E2 at added concentrations of 150, 250, and 400 pM. Because the established aptasensor calibration ranges differed between saliva and urine, different dilution schemes were used before aptasensor analysis. The saliva samples were diluted tenfold, resulting in measurement concentrations of 15, 25, and 40 pM. The urine samples fortified at 150, 250, and 400 pM were diluted 10-, 20-, and 40-fold, respectively, resulting in measurement concentrations of 15, 12.5, and 10 pM. All diluted concentrations were within the corresponding established calibration ranges. The concentrations in the original samples were subsequently obtained using the respective dilution factors.

In urine samples, the aptasensor yielded recoveries of 83.0–108.0%, with RSD values of 3.8–5.8%, whereas ELISA yielded recoveries of 92.0–101.0%, with RSD values of 4.8–8.2%. The relative biases between the two methods ranged from −9.8% to 10.2%. In saliva samples, the recoveries obtained using the aptasensor and ELISA were 85.0–112.0% and 90.0–100.0%, respectively. The corresponding RSD values were 4.0–6.3% and 5.0–8.0%, and the relative biases ranged from −5.6% to 13.1%.

The unspiked non-estrus urine and saliva samples used in this paired comparison produced responses below the detection limit of the aptasensor and below the lower quantifiable range of the ELISA kit. These samples were distinct from the pooled post-estrus urine used in the matrix-specific calibration and recovery experiments, which contained approximately 360–380 pM endogenous E2 before dilution. Overall, the relative biases for the six spiked sample groups ranged from −9.8% to 13.1%, providing preliminary evidence of agreement between the aptasensor and ELISA under the investigated spiking conditions. Detailed comparative results are provided in Table S7.

Nevertheless, this comparison represents a partial orthogonal evaluation rather than comprehensive reference-method validation across all matrices. The selected ELISA kit was not applied to milk or tap water because these matrices were not included among its specified sample types. Further validation using matrix-specific LC–MS/MS or another appropriately validated chromatographic method, together with a larger number of independently collected biological samples, will be required before routine practical application.

### 3.7. Analytical Reliability, Functional Storage Stability, and Practical Limitations

To further evaluate the analytical reliability and practical reproducibility of the proposed aptasensor, intra-day precision, inter-day reproducibility, component-batch reproducibility, preliminary robustness, operational signal stability, and short-term storage behavior were investigated.

The intra-day RSDs for spiked milk samples ranged from 10.53% to 16.11%, whereas the inter-day RSDs for spiked tap-water samples ranged from 0.89% to 3.39% (Tables S1 and S2). The intra-batch RSDs for two independently prepared AAO membrane batches were 7.47% and 7.89%, and those for two independently functionalized MNP probe batches were 2.23% and 5.03%, respectively (Table S3a,b). Small variations in pH, Mg^2+^ concentration, and incubation time yielded relative responses of 92.42–100.68%, providing preliminary evidence of method robustness (Table S5). In addition, the completed assay retained 95.58% of its initial fluorescence-ratio response over a 60-min observation period, indicating that image acquisition and signal analysis could be performed within this window without substantial signal loss (Table S6). Collectively, these results provide complementary evidence of assay precision, component-batch reproducibility, robustness, and short-term operational stability, although broader validation using additional experimental days, material batches, and biological samples remains necessary.

The short-term functional storage stability of the assembled MNP–aptamer–cDNA probe was evaluated after storage at 4 °C in the dark for up to 10 days. As summarized in Table S4, the probe retained 94.7% of its initial analytical response after 5 days and 80.0% after 10 days. Based on the retained functional response, storage for up to 5 days at 4 °C in the dark is currently recommended under the tested conditions.

It should be emphasized that this experiment evaluated the functional stability of the complete assembled sensing probe, not the stability of each component separately. Changes in nanoparticle aggregation, hydrodynamic size, ζ potential, magnetic-separation efficiency, aptamer desorption, aptamer molecular integrity, and binding affinity were not independently characterized. Therefore, the signal decrease observed after 10 days cannot be attributed exclusively to either instability of the magnetic nanoparticles or degradation of the immobilized nucleic-acid layer. Longer-term shelf-life studies combined with separate physicochemical characterization of the nanoparticles and direct evaluation of aptamer retention and binding activity will be required to establish practical storage specifications.

Despite its analytical sensitivity, the present platform should be regarded as a laboratory-scale proof of concept rather than an immediately deployable precision-livestock-monitoring system. The current workflow involves a 45-min target-recognition reaction, manual magnetic separation and washing, manual loading of the sensing complexes onto a commercial AAO membrane, coverslip assembly, dual-channel fluorescence microscopy, and image-based signal analysis. In addition, pretreatment of saliva, milk, and urine involves heating, centrifugation, dilution, and protein removal, which further increase the total sample-to-result time. No automated fluidic cartridge, portable dual-channel fluorescence reader, continuous-sampling module, or longitudinal on-farm validation was developed in the present study. Therefore, the current results demonstrate laboratory-scale analytical feasibility in pretreated samples but do not establish immediate in situ, real-time, or point-of-care monitoring capability. Future engineering efforts should focus on shortening the recognition kinetics, simplifying and standardizing sample preparation, integrating magnetic handling and AAO membrane loading into a disposable cartridge, and replacing microscope-based imaging with a portable dual-channel fluorescence reader.

The modular magnetic-separation and AAO-confinement architecture may also be evaluated in additional aqueous environmental matrices. However, the present results do not establish matrix-independent transferability or scalability. Environmental samples such as surface water, agricultural runoff, and wastewater may contain variable levels of dissolved organic matter, suspended solids, salts, metal ions, surfactants, microorganisms, and structurally related endocrine-disrupting compounds. These constituents may affect aptamer recognition, magnetic separation, fluorescence readout, or membrane loading. Application to a new environmental matrix would therefore require separate assessment of endogenous E2 and potentially cross-reactive compounds, optimization of sample pretreatment and dilution, establishment of a matrix-matched calibration or standard-addition procedure, and validation of recovery, precision, and the lower quantification limit.

## 4. Conclusions

In summary, a nanoconfinement-driven solid-state ratiometric fluorescent aptasensor was developed for E2 detection. Magnetic separation reduced matrix-derived background, while AAO nanochannel confinement improved the uniformity of the solid-state fluorescence distribution and mitigated coffee-ring-associated nonuniform deposition. However, the different calibration slopes observed in tap water, sow saliva, whole milk, and post-estrus sow urine demonstrated that residual matrix-dependent effects remained. Therefore, matrix-specific calibration is required for quantitative analysis in different sample types. Under matrix-matched calibration conditions, the assay exhibited calibration ranges of 5.0–50.0 pM in tap water and sow saliva, 5.0–40.0 pM in whole milk, and 5.0–15.0 pM in post-estrus sow urine, with an LOD of 3.62 pM in tap water. Formal residual-versus-fitted analysis and homoscedasticity testing were not performed; therefore, constant residual variance and complete calibration-model adequacy remain unverified, particularly for the preliminary post-estrus sow urine model established using only three concentration levels. A paired comparison with a commercial ELISA was performed only for spiked non-estrus sow urine and sow saliva. Tap water and whole milk were not included because these matrices were not among the sample types specified for the selected ELISA kit. Therefore, the ELISA comparison should be regarded as a partial orthogonal evaluation in two biological matrices rather than comprehensive reference-method validation across all matrices investigated in this study. Collectively, these results demonstrate the laboratory-scale analytical feasibility of the platform while also identifying the need for broader matrix-specific reference-method validation.

The aptasensor exhibited the highest response toward 17β-E2 among the tested compounds. However, E3 and 17α-E2 retained relative cross-reactivities of 77.3% and 47.3%, respectively, demonstrating that the present aptamer has limited ability to distinguish 17β-E2 from closely related natural estrogens. Therefore, the current platform should be regarded as showing preferential rather than exclusive recognition of 17β-E2. Molecular docking suggested a putative binding pose and possible noncovalent interactions between 17β-E2 and the aptamer; however, this computational model represents only a structural hypothesis because the predicted binding pose, nucleotide-level contacts, and proposed conformational interpretation were not independently validated.

The dual-color magnetic-separation and AAO-confinement architecture provides a modular laboratory framework that may be adapted to other targets by replacing the recognition aptamer. However, the current system should not be regarded as an immediately deployable in situ, real-time, or point-of-care precision-livestock-monitoring tool. The present workflow requires a 45-min target-recognition step, matrix-specific sample pretreatment, manual magnetic separation and washing, manual AAO membrane loading and slide assembly, dual-channel fluorescence microscopy, and image-based signal analysis. Although the assembled probe retained 94.7% of its initial response after 5 days at 4 °C in the dark, the response decreased to 80.0% after 10 days. Therefore, longer-term shelf-life studies and separate characterization of nanoparticle aggregation, aptamer retention, and binding activity will be required before practical storage specifications can be established. Future incorporation of recognition sequences with improved affinity and discrimination among closely related estrogens, together with shortened reaction kinetics, standardized sample pretreatment, integrated magnetic handling and membrane loading, and portable dual-channel fluorescence readout, may further improve analytical specificity, matrix tolerance, and operational simplicity. Although sample pretreatment, magnetic separation, ratiometric correction, and AAO confinement reduced matrix-associated variability, the different matrix-specific calibration slopes demonstrated that the system was not matrix independent. Transfer to additional environmental matrices will therefore require matrix-specific background assessment, pretreatment optimization, calibration or standard-addition procedures, and recovery and precision validation. Broader validation will require appropriately validated reference methods for each matrix, including LC–MS/MS or other suitable chromatographic methods for tap water, whole milk, and additional biological or environmental samples. Larger independent and longitudinal on-farm sample sets, additional steroid hormones, representative protein components, and multi-analyte coexistence conditions will also be necessary before routine on-site application. The proposed recognition model should also be experimentally evaluated through nucleotide-substitution experiments and independent biophysical or structural measurements before definitive conclusions regarding the molecular recognition mechanism can be drawn.

## Figures and Tables

**Table 1:** Oligonucleotide sequences and modifications used in this study.

Name	Sequence (5′ to 3′)	Modification	Source
E2-Aptamer	AAG GGA TGC CGT TTG GGC CCA AGT TCG GCA TAG TG	5′-Texas Red	[12]
cDNA ^1^	TTT TTT ACT TGG GCC CAA	5′−Biotin; 3′-FAM	This work

^1^ The cDNA contains a 6-mer poly-T spacer (underlined) at the 5′ end, followed by a 12-nt region fully complementary to the aptamer target region.

**Table 2:** (**a**) Analytical performance of representative recent ratiometric aptasensing platforms for 17β-estradiol detection. (**b**) Practical and operational characteristics of representative ratiometric aptasensing platforms for 17β-estradiol detection.

(**a**)								
**Platform and Ratiometric Principle**	**Linear Range**	**LOD**	**Real Sample**	**Readout Format**	**Distinctive Design Feature**	**Reference**		
MIL-53-NH_2_/Ru(bpy)_3_^2+^-silica FRET aptasensor	0.5–1000 nM	0.2 nM	Serum	Solution fluorescence	Dual-nanoprobe FRET signal referencing in homogeneous solution	[35]		
UCNP/SYBR Green I LRET integrated with μPAD	0.02–200 ng mL^−1^ (spectral); 0.05–200 ng mL^−1^ (smartphone)	NR *	Real samples	μPAD; smartphone/spectrometer	Smartphone-compatible paper readout with near-infrared-excited UCNPs	[19]		
Split-aptamer HCR with dual PEC/EC ratios	0.1–5000 pg mL^−1^ (EC); 0.1–10,000 pg mL^−1^ (PEC)	0.06 pg mL^−1^ (EC); 0.02 pg mL^−1^ (PEC)	Lake water	PEC/EC electrode	Signal amplification combined with two independent electrochemical ratios	[36]		
Label-free ethyl-violet/fluorescein ratio	NR	0.25 μM	Farm wastewater	Solution fluorescence	Label-free homogeneous dual-dye fluorescence ratio	[37]		
Split-aptamer Fc/MB@ZIF-8/LIG electrochemical ratio	10 pg mL^−1^–500 ng mL^−1^	7.60 pg mL^−1^	NR	Electrochemical DPV	Split-aptamer recognition integrated with a dual-redox electrochemical interface	[38]		
SNA DNA walker with ALP/CoOOH–g-C_3_N_4_ cascade	NR	100 pM	Water	Solution fluorescence	Enzyme/nanozyme cascade amplification with DNA-walking signal generation	[39]		
AgNC-hyperbranched DNA/metal-AIEgen FRET	10 fg mL^−1^–1 ng mL^−1^	1.2 fg mL^−1^	Real samples	Solution fluorescence	Multistage nucleic-acid amplification coupled with metal-cluster/AIE FRET	[40]		
Magnetic dual-color aptasensor with AAO confinement	5.0–50.0 pM in tap water and saliva; 5.0–40.0 pM in whole milk; 5.0–15.0 pM in urine	3.62 pM (tap water)	Tap water, sow saliva, whole milk, and post-estrus sow urine	AAO-assisted solid-state dual-color fluorescence ratio	Magnetic matrix cleanup combined with dual-color referencing and AAO-assisted spatial localization of the solid-state signal	This work		
(**b**)								
**Platform**	**Total Analysis Time**	**Washing**	**Stability**	**Estimated Cost**	**Preparation**	**Reusability**	**Instrumentation**	**References**
MIL-53-NH_2_/Ru-silica FRET	NR	None during assay	NR	High	Complex	ND	Moderate	[35]
UCNP–μPAD LRET	NR	None during assay	NR	Moderate–high	Complex	No	Low–moderate	[19]
Split-aptamer HCR PEC/EC	NR	Multiple	NR	High	Complex	ND	High	[36]
Ethyl-violet/fluorescein	NR	None	NR	US $0.11/assay	Simple	ND	Moderate	[37]
Fc/MB@ZIF-8/LIG	NR	Multiple	NR	Moderate–high	Complex	ND	Moderate	[38]
SNA DNA walker cascade	NR	None during assay	NR	High	Very complex	ND	Moderate	[39]
AgNC/metal-AIEgen FRET	NR	None during assay	NR	High	Very complex	ND	Moderate	[40]
This work	≥45 min plus post-recognition handling	2 magnetic washes	94.7% at 5 d; 80.0% at 10 d (4 °C, dark)	Moderate	Moderate	ND	High	This work

**Notes**: * NR, not reported or not reliably extractable from the available source information. Concentration units and analytical ranges are retained as reported in the original studies to avoid errors caused by unit conversion. Analysis time excludes matrix-specific sample pretreatment and off-line probe/device preparation unless otherwise stated. Estimated cost includes disposable reagents and sensing materials but excludes labor and capital instrumentation. Detailed criteria for preparation and instrumental complexity are provided in Table S8. ND, not demonstrated. All other abbreviations are defined in the Abbreviations section at the end of the manuscript.

## Data Availability

The original contributions presented in this study are included in the article/Supplementary Materials. Further inquiries can be directed to the corresponding author.

## Supplementary Materials

The following supporting information can be downloaded at https://www.mdpi.com/article/10.3390/bios16080419/s1: Table S1: Intra-day precision; Table S2: Inter-day reproducibility; Table S3: (a): Reproducibility across two independently prepared AAO membrane batches. (b): Reproducibility across two independently functionalized MNP probe batches; Table S4: Short-term functional storage stability; Table S5: Preliminary robustness assessment; Table S6: Operational signal stability; Table S7: Paired aptasensor–ELISA comparison; Table S8: Detailed basis for the practical comparison of representative ratiometric aptasensing platforms; Table S9: Calibration parameters and regression statistics; Table S10: (a): Fluorescence-based quantification of cDNA-FAM immobilization efficiency across 59 independently prepared MNP probe batches, (b) luorescence-based quantification of apparent Texas Red-aptamer retention after the 67 initial post-hybridization washing steps across independently prepared MNP probe batches, (c) Summary of cDNA-FAM immobilization and apparent Texas Red-aptamer retention 78 before the final stringent washing steps; Table S11: Individual cross-reactivity and competitive interference results.

## Abbreviations

The following abbreviations are used in this manuscript:

AAO	Anodic aluminum oxide
AgNC	Silver nanocluster
AIEgen	Aggregation-induced emission luminogen
ALP	Alkaline phosphatase
cDNA	Complementary DNA
DPV	Differential pulse voltammetry
E2	17β-estradiol
E3	Estriol
EC	Electrochemical
ELISA	Enzyme-linked immunosorbent assay
FAM	Carboxyfluorescein
Fc	Ferrocene
FE-SEM	Field-emission scanning electron microscope
FRET	Förster resonance energy transfer
HCR	Hybridization chain reaction
HRTEM	High-resolution transmission electron microscopy
LC-MS/MS	Liquid chromatography-tandem mass spectrometry
LIG	Laser-induced graphene
LOD	Limit of detection
LRET	Luminescence resonance energy transfer
MB	Methylene blue
MNP	Magnetic nanoparticle
μPAD	Microfluidic paper-based analytical device
PBS	Phosphate-buffered saline
PEC	Photoelectrochemical
PLF	Precision livestock farming
SA-MNP	Streptavidin-coated magnetic nanoparticle
SELEX	Systematic evolution of ligands by exponential enrichment
SNA	Spherical nucleic acid
TEM	Transmission electron microscopy
TISD	Target-induced strand displacement
UCNP	Upconversion nanoparticle
ZIF-8	Zeolitic imidazolate framework-8
