# Supplementary material for: Nanoconfinement-Driven Solid-State Ratiometric Fluorescent Aptasensor for 17β-Estradiol Detection in Complex Matrices
Source: Biosensors (Basel). 2026 Aug 3;16(8):419. doi: 10.3390/bios16080419 (PMC13511469; doi:10.3390/bios16080419)
Supplement: Supplementary file 1 [file biosensors-16-00419-s001.zip › biosensors-4426185-supplementary.pdf]

biosensors

MDPI

Supplementary

Nanoconfinement-Driven Solid-State Ratiometric Fluorescent Aptasensor for 17β-Estradiol Detection in Complex Matrices

Shanshan Zheng, Hui Wang *, Zhixue Yu, Ruipeng Chen, Liang Yang, Benhai Xiong and Xiangfang Tang

State Key Laboratory of Animal Nutrition and Feeding, Institute of Animal Science, Chinese Academy of Agricultural Sciences, Beijing 100193, China; zhengshanshan@caas.cn (S.Z.)
* Correspondence: wanghui01@caas.cn; Tel.: +86-10-6281-6017

Table S1. Intra-day precision.

Matrix	E2 added	n	Found concentration (pM)	Mean	SD	RSD	Recovery
Milk	5pM	5	5.34,4.80,4.23,5.22,5.87	5.092	0.615	12.07%	101.84%
Milk	20pM	5	23.28,16.02,18.94,20.89,16.34	19.09	3.08	16.11%	95.47%
Milk	40pM	5	33.51,41.57,43.28,44.49,41.69	40.91	4.31	10.53%	102.27%

Table S2. Inter-day reproducibility.

Matrix	E2 concentration	Day 1 ΔRatio, n=3	Day 2 ΔRatio,n=3	Overall Mean	SD	Inter-day RSD
Water	5pM	0.31578	0.30109	0.308435	0.01039	3.37%
Water	20pM	1.28997	1.22961	1.25629	0.04269	3.39%
Water	40pM	1.60882	1.5888	1.59881	0.01415	0.89%

Table S3a. Reproducibility across two independently prepared AAO membrane batches.

AAO batch	E2 concentration	n	ΔRatio (individual membranes)	Mean ΔRatio	SD	Intra-batch RSD
AAO-1	10pM	3	0.67511,0.63049,0.58122	0.6289	0.0470	7.47%
AAO-2	10pM	3	0.59336,0.62478,0.69193	0.6367	0.0502	7.89%

Note: The inter-batch RSD calculated from the mean responses of the two independently prepared AAO membrane batches was 0.87%, with a relative percent difference of 1.23%.

Table S3b. Reproducibility across two independently functionalized MNP probe batches.

MNP probe batch	E2 concentration	n	ΔRatio	Mean ΔRatio	SD	Intra-batch RSD
MNP-1	20pM	3	1.26823,1.25687,1.21541	1.2468	0.0278	2.23%
MNP-2	20pM	3	1.32135,1.28690,1.41562	1.3413	0.0675	5.03%

Note: The inter-batch RSD calculated from the mean responses of the two independently functionalized MNP probe batches was 5.16%, with a relative percent difference of 7.30%.

Table S4. Short-term functional storage stability of the assembled magnetic aptamer probe at 4 °C in the dark.

Storage time	E2 concentration	ΔRatio	Mean ΔRatio	SD	Response retention (%)	Change from Day 0 (%)	RSD

Day 0	20pM	1.26823,1.25687,1.21541	1.2468	0.0278	100.0	0	2.23%
Day 5	20pM	1.18711,1.14956,1.20733	1.1813	0.0293	94.7	-5.3	2.48%
Day 10	20pM	1.05276,1.03545,0.90412	0.9974	0.0801	80.0	-20.0	8.03%

Note: Data are presented as mean ± SD (n = 3). The experiment evaluated the analytical response of the complete MNP–aptamer–cDNA probe and did not separately assess nanoparticle colloidal stability or aptamer molecular integrity.

Table S5. Preliminary robustness assessment of the proposed aptasensor under small variations in experimental conditions.

Parameter	Nominal condition	Tested condition	n	Individual ΔRatio	Mean ΔRatio	SD	RSD	Relative response (%)
pH	7.4	7.2	3	1.1821,1.1537,1.1211,	1.1523	0.0305	2.65	92.42
pH	7.4	7.4	3	1.26823,1.25687,1.21541	1.2468	0.0278	2.23	100
pH	7.4	7.6	3	1.2317,1.1995,1.1762	1.2025	0.0279	2.32	96.44
Mg^{2+} concentration	5mM	4.5 mM	3	1.2179,1.3461,1.1018	1.2219	0.1222	10.00	98.00
Mg^{2+} concentration	5mM	5.0 mM	3	1.26823,1.25687,1.21541	1.2468	0.0278	2.23	100
Mg^{2+} concentration	5mM	5.5mM	3	1.2278,1.2526,1.2855	1.2553	0.0289	2.31	100.68
Incubation time	45min	40 min	3	1.1926,1.1838,1.2143	1.1969	0.0157	1.31	95.99
Incubation time	45min	45 min	3	1.26823,1.25687,1.21541	1.2468	0.0279	2.23	100
Incubation time	45min	50 min	3	1.2638,1.2176,1.2792	1.2535	0.0321	2.56	100.54

Note: Robustness was evaluated using 20 pM E2 in buffer. All measurements were performed in triplicate (n = 3). When one experimental parameter was varied, all other parameters were maintained at their optimized conditions. The mean ΔRatio obtained under the nominal optimized condition (pH 7.4, 5.0 mM Mg^{2+}, and 45 min incubation) was defined as 100%. SD was calculated using the sample standard deviation.

Table S6. Time-dependent stability of the fluorescence-ratio response after assay completion.

Time after assay completion (min)	n	Individual ΔRatio	Mean ΔRatio	SD	RSD	Relative response (%)	Change from initial response (%)
0	3	1.2682,1.2568,1.2154	1.2468	0.0278	2.23	100	0.00
10	3	1.2436,1.2283,1.2591	1.2437	0.0154	1.24	99.75	-0.25
20	3	1.2512,1.2331,1.2367	1.2403	0.0096	0.77	99.48	-0.52
30	3	1.2117,1.2145,1.2383	1.2215	0.0146	1.20	97.97	-2.03
45	3	1.1995,1.2147,1.2079	1.2074	0.0076	0.63	96.84	-3.16
60	3	1.1876,1.2015,1.1858	1.1916	0.0086	0.72	95.58	-4.42

Note: Operational signal stability was evaluated using 20 pM E2 in buffer under the optimized conditions. Three independently prepared sensing samples were tested (n = 3), and the same samples were repeatedly measured at predetermined time points after completion of the sensing reaction. The mean ΔRatio obtained at 0 min was defined as 100%. Samples were protected from light between measurements to minimize photobleaching. SD was calculated using the sample standard deviation. The 0-min data correspond to the nominal-condition control presented in Table A5.

Table S7. Paired comparison of the aptasensor and ELISA for E2 determination in spiked non-estrus sow urine and sow saliva.

Matrix	E2 added (pM)	Dilution factor	Concentration after dilution(pM)	Aptasensor result (pM, mean ± SD)	Aptasensor RSD (%)	ELISA result (pM,	ELISA RSD (%)	Aptasensor recovery (%)	ELISA recovery (%)	Relative bias (%)

					mean ± SD)					
Non-estrus sow urine	0	10	0	< LOD	—	< LLOQ	—	—	—	—
Non-estrus sow urine	150	10	15	124.5 ± 7.2	5.80%	138.0 ± 11.3	8.20%	83.00%	92.00%	-9.80%
Non-estrus sow urine	250	20	12.5	270.0 ± 11.3	4.20%	245.0 ± 13.5	5.50%	108.00%	98.00%	10.20%
Non-estrus sow urine	400	40	10	368.0 ± 14.0	3.80%	404.0 ± 19.4	4.80%	92.00%	101.00%	-8.90%
Saliva	0	10	0	< LOD	—	< LLOQ	—	—	—	—
Saliva	150	10	15	127.5 ± 8.0	6.30%	135.0 ± 10.8	8.00%	85.00%	90.00%	-5.60%
Saliva	250	10	25	280.0 ± 12.6	4.50%	247.5 ± 13.6	5.50%	112.00%	99.00%	13.10%
Saliva	400	10	40	380.0 ± 15.2	4.00%	400.0 ± 20.0	5.00%	95.00%	100.00%	-5.00%

Note: The urine used for this comparison was collected from non-estrous sows and was distinct from the pooled post-estrus sow urine used for the matrix-specific calibration and spike-recovery evaluation in Figure 10. Before aptasensor analysis, saliva samples were diluted tenfold, whereas urine samples containing 150, 250, and 400 pM added E2 were diluted 10-, 20-, and 40-fold, respectively. The concentrations in the original samples were calculated using the corresponding dilution factors. Each independently prepared ELISA sample was measured in duplicate wells, and the duplicate wells were averaged as technical replicates. The unspiked non-estrus urine and saliva samples were below the detection limit of the aptasensor and below the lower quantifiable range of the ELISA kit.

Table S8. Detailed basis for the practical comparison of representative ratiometric aptasensing platforms for 17β-estradiol detection.

Platform	Total analysis time and washing	Stability	Estimated cost and basis	Preparation and reusability	Instrumental complexity	Ref.
MIL-53-NH_2/$Ru(bpy)_3^{2+}$-silica FRET aptasensor	Total sample-to-result time: NR. No post-recognition washing was reported for the homogeneous assay; off-line preparation of the two nanoprobes requires purification/washing.	NR	High (qualitative): two functionalized nanoprobes, modified oligonucleotides, MOF and Ru-complex materials; labor and capital equipment excluded.	Complex: separate donor/acceptor probe preparation and aptamer–cDNA assembly. Reusability was not demonstrated.	Moderate: dual-emission fluorescence spectrometer.	[35]

UCNP/SYBR Green I LRET–μPAD platform	Total sample-to-result time: NR. No routine post-recognition wash was reported; UCNP functionalization and μPAD fabrication require off-line purification/assembly.	NR	Moderate–high (qualitative): multistep UCNP synthesis and paper-device preparation; near-infrared excitation remains necessary.	Complex: UCNP synthesis, nucleic-acid functionalization and μPAD assembly. Disposable, not reusable.	Low–moderate: smartphone with compact near-infrared excitation; spectrometer optional.	[19]
Split-aptamer HCR with dual PEC/EC ratios	Total sample-to-result time: NR. Recognition, HCR amplification and sequential PEC/EC measurement are required, with multiple electrode rinsing/washing steps.	NR	High (qualitative): CdTe QDs, multiple DNA strands, HCR reagents, electrode materials and dual-mode measurement.	Complex: electrode modification, split-aptamer assembly and HCR amplification. Reuse was not demonstrated.	High: electrochemical workstation plus photoelectrochemical excitation.	[36]
Label-free ethyl-violet/fluorescein aptasensor	Total sample-to-result time: NR. No washing is required during routine homogeneous measurement.	NR	Approximately US$0.11 per assay, as reported by the authors.	Simple: direct mixing of commercial dyes, aptamer and sample. Reuse was not demonstrated.	Moderate: fluorescence spectrometer with two emission bands.	[37]
Split-aptamer Fc/MB@ZIF-8/LIG electrochemical aptasensor	Total sample-to-result time: NR. Electrode fabrication and bioreceptor immobilization involve multiple rinsing steps; routine analysis requires target incubation and electrochemical measurement.	Quantitative storage-retention data: NR.	Moderate–high (qualitative): laser-induced graphene, MB@ZIF-8, Fc-labeled oligonucleotides and electrode reagents.	Complex: laser fabrication, composite deposition and split-aptamer assembly. Reuse was not demonstrated.	Moderate: electrochemical workstation.	[38]
SNA DNA walker with ALP/CoOOH–g-C_3N_4 cascade	Total sample-to-result time: NR. No final wash is required, but sequential DNA-walking, enzymatic and redox reactions are involved.	NR	High (qualitative): SNA/AuNP probes, several DNA strands, restriction enzyme, ALP, CoOOH and g-C_3N_4.	Very complex: multicomponent nanoprobe preparation and coupled enzyme/nanozyme amplification. Reuse was not demonstrated.	Moderate: fluorescence spectrometer and controlled enzymatic incubation.	[39]
AgNC-hyperbranched DNA/metal-AIEgen FRET platform	Total sample-to-result time: NR. No final wash was reported; preparation of metal-AIEgen frameworks, AgNC probes and amplified DNA structures requires off-line synthesis/purification.	NR	High (qualitative): metal-AIEgen frameworks, AgNC reagents, multiple designed DNA strands and amplification components.	Very complex: multistep material synthesis and nucleic-acid amplification. Reuse was not demonstrated.	Moderate: fluorescence spectrometer with temperature-controlled reactions.	[40]
Magnetic dual-color aptasensor with AAO confinement (this work)	Approximately 55–65 min after matrix-specific pretreatment: 45-min recognition, magnetic separation,	Probe response retained 94.7% after 5 d and	Moderate (qualitative): magnetic beads, two labeled oligonucleotides, AAO	Moderate: commercial beads and AAO are used, but sequential oligonucleotide	High: thermomixer, magnetic rack, dual-channel fluorescence microscope, CCD and image analysis.	This work

https://doi.org/10.3390/bios16080419

two washes, AAO loading, dual-channel imaging and image analysis. Off-line probe preparation is excluded.	80.0% after 10 d at 4 °C in the dark; completed-assay signal retained 95.58% over 60 min.	membrane, buffers and disposables; labor and capital instrumentation excluded.	assembly, 12–16 h hybridization and repeated off-line magnetic washing are required. Reuse was not demonstrated.

Notes: Total analysis time excludes off-line probe/device fabrication and matrix-specific sample pretreatment unless stated.
Cost ratings are qualitative estimates of disposable reagents and sensing materials; labor and capital instrumentation are excluded. Exact monetary values are retained only when reported by the original authors.
Preparation: simple, direct mixing; moderate, limited assembly using mainly commercial components; complex, multiple fabrication/functionalization steps; very complex, coupled material and amplification processes.
Instrumental complexity: low, portable visual/smartphone readout; moderate, one routine fluorescence/electrochemical reader; high, multiple modules or microscope-based dual-channel imaging. NR, not reported.

Table S9. Matrix-specific calibration statistics for 17β-estradiol determination.

Matrix	Baseline Ratio$_0$	Calibration range	Concentration levels	Regression equation	R^2	SE of slope	95% CI of slope
Tap Water	3.663 ± 0.089	5-50pM	5,10,25,40,50	y=0.0591+0.520x	0.9988	0.010	0.487-0.553
Sow Saliva	2.598 ± 0.114	5-50pM	5,10,20,40,50	y=−0.000417+0.363x	0.9926	0.018	0.305-0.420
Whole Milk	1.297 ± 0.096	5-40pM	5,10,20,40	y=0.0555+0.252x	0.9935	0.014	0.190-0.314
Sow Urine	0.933 ± 0.056	5-15pM	5,10,15	y=0.0664+0.172x	0.9999	0.001	0.160-0.184

Note: $x=\log_{10}(C_{E2}/pM)$. SE, standard error; CI, confidence interval. The slope standard errors and 95% confidence intervals are reported as estimates of regression-parameter uncertainty. Formal residual-versus-fitted analysis and homoscedasticity testing were not performed; therefore, random residual distribution, constant residual variance, and complete calibration-model adequacy were not independently verified. The post-estrus sow urine model should be regarded as preliminary because it was established using only three concentration levels.

Table S10a. Fluorescence-based quantification of cDNA-FAM immobilization efficiency across independently prepared MNP probe batches.

Batch	Total cDNA-FAM added (pmol)	Unbound cDNA-FAM in pooled supernatant + washes (pmol)	Immobilized cDNA-FAM (pmol)	Immobilization efficiency (%)
1	1200	234	966	80.5
2	1200	261	939	78.3
3	1200	213	987	82.3
**Mean**	**1200**	**236 ± 24**	**964 ± 24**	**80.4 ± 2.0**
**RSD (%)**	—	**10.2**	**2.5**	**2.5**

https://doi.org/10.3390/bios16080419

Note: The immobilization efficiency was calculated as (immobilized cDNA-FAM / total cDNA-FAM added) × 100%. The unbound cDNA-FAM was determined by pooling all supernatant and wash fractions from the three washing steps. Data represent the mean ± SD from three independently prepared MNP probe batches. The immobilization efficiencies ranged from 78.3% to 82.3% across batches with an RSD of 2.5%, indicating that the streptavidin–biotin anchoring strategy enables reproducible cDNA-FAM immobilization. The slightly higher RSD for the unbound fraction (10.2%) is attributed to batch-to-batch variations in washing efficiency, which did not significantly compromise the overall reproducibility of immobilization.

Table S10b. Fluorescence-based quantification of apparent Texas Red-aptamer retention after the initial post-hybridization washing steps across independently prepared MNP probe batches.

Batch	Total Texas Red-aptamer added (pmol)	Unbound Texas Red-aptamer in pooled supernatant + washes (pmol)	Texas Red-aptamer retained after the initial washing steps (pmol)	Apparent retention efficiency (%)	Apparent retained aptamer/cDNA-FAM molar ratio
1	350	32	318	90.9	0.329
2	350	41	309	88.3	0.329
3	350	27	323	92.3	0.327
**Mean**	**350**	**33 ± 7**	**317 ± 7**	**90.5 ± 2.0**	**0.328 ± 0.001**
**RSD (%)**	—	**21.2**	**2.2**	**2.2**	**0.3**

Note: The apparent retention efficiency was calculated as the amount of Texas Red-labeled aptamer remaining associated with the MNP–cDNA complexes after the initial three post-hybridization washing steps divided by the total aptamer amount initially added. The unbound aptamer was determined from the pooled post-hybridization supernatant and these three initial washing fractions. The subsequent thermal-equilibration step and five stringent washing steps were not included in the fluorescence mass-balance measurement. Therefore, the reported values represent apparent aptamer retention at an intermediate stage of probe preparation and should not be interpreted as the final aptamer-loading efficiency of the ready-to-use probe. Data are presented as mean ± SD from three independently prepared MNP probe batches. The apparent retained aptamer/cDNA-FAM molar ratio of 0.328 ± 0.001 was consistent with the intended substoichiometric aptamer input, leaving an excess of immobilized cDNA relative to the retained reporter aptamer.

Table S10c. Summary of cDNA-FAM immobilization and apparent Texas Red-aptamer retention before the final stringent washing steps.

Parameter	Value per batch	RSD（%）	Value per mg MNP
cDNA-FAM immobilized	964 ± 24 pmol	2.5	964 ± 24 pmol mg^{-1}
Texas Red-aptamer retained after the initial washing steps	317 ± 7 pmol	2.2	317 ± 7 pmol mg^{-1}
Apparent retained Apt/cDNA ratio before stringent washing	0.328 ± 0.001	0.3	0.328 ± 0.001

Note: The Texas Red-aptamer values were calculated before the subsequent thermal-equilibration and five stringent washing steps and therefore represent intermediate apparent retention values rather than final loading values of the ready-to-use probe. The estimated molecules per bead are theoretical calculations based on assumed particle diameter, density, spherical geometry, and uniform probe distribution; they were not directly measured at the single-bead level.

Table S11. Quantitative analysis of individual cross-reactivity and competitive interference effects of potential interferents toward 17β-E2 detection.

Group	Added interferent(s) (50 pM each)	ΔRatio (mean ± SD, n=5)	Relative response (%)*	p value vs. E2-only**
Blank	None (0 pM E2)	0.001 ± 0.005	—	—
E2 only	None	0.190 ± 0.009	100.0	—
E2 + 17α-E2	17α-E2	0.164 ± 0.012	86.3	< 0.05
E2 + E3	E3	0.148 ± 0.014	77.9	< 0.01
E2 + BPA	BPA	0.187 ± 0.010	98.4	> 0.05 (n.s.)
E2 + Mix	17α-E2 + E3 + BPA	0.139 ± 0.015	73.2	< 0.001

Note: Data are presented as mean ± SD of five independently prepared assays (n = 5). For individual cross-reactivity evaluation, 50 pM of each analyte (17β-E2, 17α-E2, E3, and BPA) was tested, and relative cross-reactivity was calculated by normalizing the ΔRatio response of each interferent to that of 50 pM 17β-E2. For competitive interference evaluation, 10 pM 17β-E2 was analyzed alone or in the presence of a fivefold molar excess (50 pM) of 17α-E2, E3, BPA, or a mixture of all interferents. The relative response was calculated by defining the response of 10 pM 17β-E2 alone as 100%. Statistical significance was evaluated using one-way ANOVA followed by Tukey's multiple-comparison test.

https://doi.org/10.3390/bios16080419
